# Evaluation of Phytochemistry and Pharmacological Properties of *Alnus nitida*

**DOI:** 10.3390/molecules27144582

**Published:** 2022-07-18

**Authors:** Moniba Sajid, Muhammad Rashid Khan, Muhammad Umar Ijaz, Hammad Ismail, Muhammad Zeeshan Bhatti, Sayed Afzal Shah, Saima Ali, Muhammad Usman Tareen, Saqer S. Alotaibi, Sarah M. Albogami, Gaber El-Saber Batiha

**Affiliations:** 1 Department of Biochemistry, Faculty of Biological Sciences, Quaid-i-Azam University, Islamabad 45320, Pakistan; moniba_qau@yahoo.com (M.S.); mrkhanqau@yahoo.com (M.R.K.); saima.ali@bs.qau.edu.pk (S.A.); sunnytareen@hotmail.com (M.U.T.); 2 Department of Zoology, Wildlife and Fisheries, University of Agriculture, Faisalabad 38040, Pakistan; umar.ijaz@uaf.edu.pk; 3 Department of Biochemistry and Biotechnology, University of Gujrat, Gujrat 50700, Pakistan; 4 Department of Biological Sciences, National University of Medical Science, Rawalpindi 46000, Pakistan; afzal.shah@numspak.edu.pk; 5 Department of Biotechnology, College of Science, Taif University, P.O. Box 11099, Taif 21944, Saudi Arabia; saqer@tu.edu.sa (S.S.A.); dr.sarah@tu.edu.sa (S.M.A.); 6 Department of Pharmacology and Therapeutics, Faculty of Veterinary Medicine, Damanhour University, Damanhour 22511, AlBeheira, Egypt; dr_gaber_batiha@yahoo.com

**Keywords:** analgesic, anti-inflammatory, *Alnus nitida*, GC-MS, pharmacological activities

## Abstract

In the current study, the anti-inflammatory and analgesic potential of *Alnus nitida* (leaves and fruits) was evaluated in the Sprague-Dawley rat. Traditionally, *A. nitida* was used for the treatment of inflammatory ailments. However, *A. nitida* leaves and fruits have not been yet reported regarding any potential medicinal effects. Leaves/fruits of *A. nitida* were extracted with methanol and fractionated to attain n-hexane, chloroform, ethyl acetate and aqueous fractions. These extracts were then evaluated for in vivo analgesic and anti-inflammatory potential. For in vivo anti-inflammatory activity, carrageenan-induced paw edema assay, Freunds’ complete adjuvant-induced edema, xylene-induced ear edema and histamine-induced paw edema models were used in rats, which showed significant (*p* < 0.01) reduction (70–80%) in edema in comparison of inflammatory controls. On other hand, for the analgesic assessment, hot plate assay and acetic acid-induced writhing tests were used, which showed a significant (*p* < 0.01) rise in latency time (40–60%) as compared with pain-induced controls. These results were comparable with standard drugs in a concentration-dependent manner and no mortality or toxicity was observed during all experiments. Then, for the identification of chemical constituents gas chromatography–mass spectrometry (GC-MS) analysis was performed, which indicated the presence of neophytadiene, 3,7,11,15-Tetramethyl-2-hexadecen-1-ol, phytol and vitamin E, justifying the use of *A. nitida* to treat inflammatory disorders.

## 1. Introduction

Although inflammation is a protective response of the body, it can also cause various degrees of injury, including allergic reactions, edema, effusion and scarring. Currently major global health concerns include inflammatory diseases, such as arthritis, cardiovascular disease, diabetes mellitus and others [1,2]. The inflammatory process involves a series of events that can be elicited by numerous stimuli, for example, infection agents, ischemia, antigen–antibody interactions and chemical, thermal or mechanical injury [3]. Several animal models were employed in this study to assess the anti-inflammatory effects and the rationale behind utilizing different model is that each model represents distinct mechanistic approach. The carrageenan-induced acute inflammatory process in rats comprises three phases: the first phase releases histamine and serotonin; the second phase is mediated by kinins; and the final phase is mediated by prostaglandins [4]. Therefore, it appears to cause inflammation by a different mechanism than that of substances like xylene, which causes an allergic response to dextran. When xylene is administered topically, a neurogenic inflammation is induced. The molecular mechanisms governing the production of pro-inflammatory chemicals from sensory neurons are mainly highlighted by this inflammation model [5]. Likewise, histamine induces the activation of microglia in vivo and prompts proinflammatory cytokine release, which suggests that histamine plays an important role in microglia activation and neuroinflammation-related diseases [6]. On the other hand, adjuvant-induced arthritis is developed after induction of complete Freund’s adjuvant into rats [7]. It is a systemic disease with articular and visceral manifestation that resembles rheumatoid arthritis. It is characterized by chronic evolution with recurrent inflammatory bouts resulting in periarticular, articular and bone lesion [4].

Pain is the most common symptom associated with minor ailments to life-threatening conditions. Analgesic agents are drugs that release pain without disturbing its cause, including non-steroidal anti-inflammatory drugs (NSAIDs) and antidepressants [8]. NSAIDs are among the most broadly used of all therapeutic classes of drugs because they are both anti-inflammatory and analgesic. Analgesic opioids are generally comprised of sulfentanil, codeine, fentanyl, morphine, methadone and meperidine. However, all these come with side effects like gastrointestinal and renal injuries [9,10,11]. Few NSAIDs have been associated with augmented blood pressure, the increased danger of congestive heart failure and the existence of thrombosis [12]. Two different analgesic testing methods were used in the current investigation to identify possible peripheral and central analgesic effects of extracts. To evaluate for a possible central anti-nociceptive effect, the hot plate test was used while for peripheral anti-nociceptive effects, the acetic acid-induced writhing test was employed.

Natural anti-inflammatory and analgesic agents based on plants are growing in popularity because of their minimal or non-existent side effects. The expansion of pharmacological mediators has been a chief focus of analgesic- and inflammatory-associated study regarding the outlook of their mass from a biomedicinal perspective. Therefore, the search for complementary and alternative sources has become increasingly vital to develop innovative types of natural ethnomedicinal drugs. Medicinal plants have been a chief reservoir of variety of biochemical compounds for several centuries and have been consumed broadly in raw or purified form to cure different ailments, including inflammation [13]. The use of plants and their parts and extracts as anti-inflammatory agents is widespread in many geographical areas. *Atriplex vesicaria* is used to treat inflammatory conditions and painful disorders [14]. The anti-inflammatory action of *Salvia sagittata* ethanolic extracts, and *Zanthoxylum myriacanthum* var. pubescens bark extracts has been documented [15,16]. Similarly, in Pakistan, various plants, including *Aristolochia indica*, *Melilotus indicus*, *Tribulus terrestris* and *Cuscuta pedicellata*, exhibit anti-inflammatory properties [17].

*A. nitida* (spach)-Endl. of the family Betulaceae is found in the Western Himalayas. In Punjab, its local name is Sharoliin, whereas in Kashmir it is known as Seril [18,19]. *Alnus* species contain diaryl heptanoids of 1,7-diphenylheptane skeleton and are reported to have different healing effects, such as antioxidant, anti-inflammatory and antitumor. About four hundred diarylheptanoids [20] have been identified from various species of *Alnus* depicting numerous pharmacological actions: anti-inflammatory [21,22], anti-influenza [23], hepatoprotective [24], etc. From *A. nitida*, two diarylheptanoids nitidone A and nitidone B have been isolated [25].

The leaves of *A. nitida* (spach)-Endl. are usually used as anti-inflammatory agents by local practitioners. They usually warm the leaves to cure boils and swellings [26]. Previously, we reported on the bark of *A. nitida* as a potential candidate for injuries and swelling [27]. However, *A. nitida* leaves have not been yet reported for any potential medicinal effects. Therefore, in the current study, we evaluated the anti-inflammatory and analgesic perspective of *A. nitida* leaves and fruit. The GC-MS analysis of *A. nitida* leaves and fruits extracts was carried out to identify the biochemical compounds responsible for these therapeutic effects.

## 2. Results

### 2.1. Acute Toxicity Studies

The animals did not show any kind of abnormal behaviors, such as water and food intake, attention, locomotion, appetite, analgesia, writhing, nasal release, aggressiveness, posture and urination and neither exhibited any change in the death rate.

### 2.2. Assessment of Anti-Inflammatory Activity 

#### 2.2.1. Carrageenan-Induced Paw Edema

**Figure 1:**
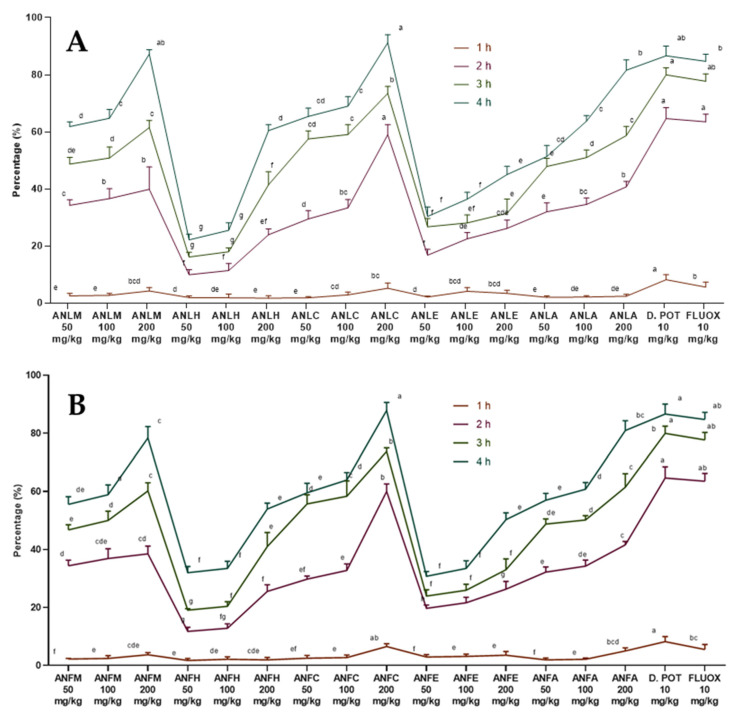
Percentage inhibition of *A. nitida* on carrageenan-induced paw edema in rats (**A**) *A. nitida* leaves methanolic extract (ANLM), *A. nitida* leaves n-hexane fraction (ANLH), *A. nitida* leaves chloroform fraction (ANLC), *A. nitida* leaves ethyl acetate fraction (ANLE), *A. nitida* leaves aqueous fraction (ANLA), and (**B**) *A. nitida* fruit methanolic extract (ANFM), *A. nitida* fruit n-hexane fraction (ANFH), *A. nitida* fruit chloroform fraction (ANFC), *A. nitida* fruit ethyl acetate fraction (ANFE), *A. nitida* fruit aqueous fraction (ANFA). D.POT: Diclofenac potassium; FLUOX: Fluoxetine. Data were analyzed using seven replicates as mean ± SD by a two-way ANOVA followed by Bonferroni’s test. Different letters represent the significance level of *p* < 0.05 with respect to all other samples “a, b” with reference to positive controls, “c” with reference to methanolic extract and “d, e, f, g” with reference to n-hexane, chloroform, ethyl acetate and aqueous fractions, respectively.

The extracts/fractions of *A. nitida* leaves and fruits showed a dose- and time-dependent decline in edema (Table S1 in the Supplementary Materials). The percentage activity is presented in Figure 1. The activity became significant (*p* < 0.01) at the lowest dose after 2 h of carrageenan injection while edema was strongly inhibited after 4 h of injection. The *A. nitida* leaves chloroform fraction (ANLC) and *A. nitida* leaves methanolic extract (ANLM) at the highest dose decreased the edema to 91.23 ± 2.80% and 87.23 ± 1.52%, whereas diclofenac potassium and fluoxetine showed 86.63 ± 3.42% and 84.71 ± 2.46% inhibition after 4 h of injection (Figure 1A and Table S1). Likewise, in fruit ANFC after 4 h showed 87.86 ± 2.78% inhibition of edema (Figure 1B and Table S2).

#### 2.2.2. Freunds’ Complete Adjuvant Induced Arthritis

**Figure 2:**
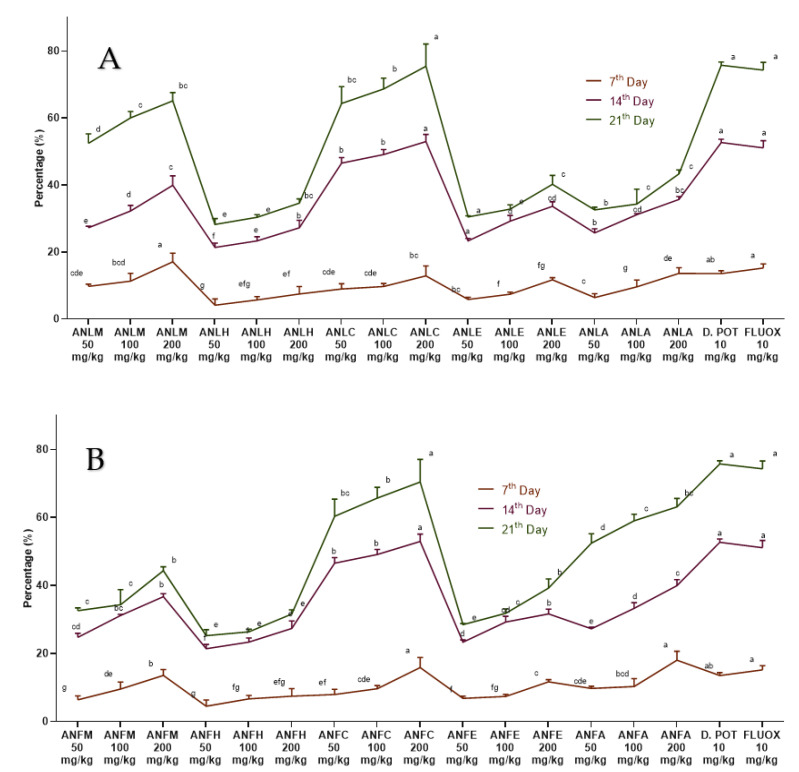
Percentage inhibition of *A. nitida* on Freunds’ complete adjuvant induced arthritis. (**A**) *A. nitida* leaves methanolic extract (ANLM), *A. nitida* leaves n-hexane fraction (ANLH), *A. nitida* leaves chloroform fraction (ANLC), *A. nitida* leaves ethyl acetate fraction (ANLE), *A. nitida* leaves aqueous fraction (ANLA), and (**B**) *A. nitida* fruit methanolic extract (ANFM), *A. nitida* fruit n-hexane fraction (ANFH), *A. nitida* fruit chloroform fraction (ANFC), *A. nitida* fruit ethyl acetate fraction (ANFE), *A. nitida* fruit aqueous fraction (ANFA). D.POT: Diclofenac potassium; FLUOX: Fluoxetine. Data were analyzed using seven replicates as mean ± SD by two-way ANOVA followed by Bonferroni’s test. Different letters represent the significance level of *p* < 0.05 with respect to all other samples “a, b” with reference to positive controls, “c” with reference to methanolic extract and “d, e, f, g” with reference to n-hexane, chloroform, ethyl acetate and aqueous fraction, respectively.

The observations obtained from this test are reported in Figure 2 and Tables S3 and S4. After edema induction (14 days), ANLC and *A. nitida* fruit chloroform fraction (ANFC) at all doses (50, 100 and 200 mg/kg) showed markedly (*p* < 0.01) decreased edema. The edema was further significantly (*p* < 0.01) repressed after 21 days by chloroform, methanol and aqueous fractions of *A. nitida* leaves and fruit. However, multiple comparisons of many treatments showed that ANLC and ANFC at 200 mg/kg almost equally decreased in edema, as shown by diclofenac potassium and fluoxetine. However, the decrease in edema of these dosages was considerably higher (*p* < 0.01) relative to other samples.

#### 2.2.3. Histamine Induced Paw Edema Method

**Figure 3:**
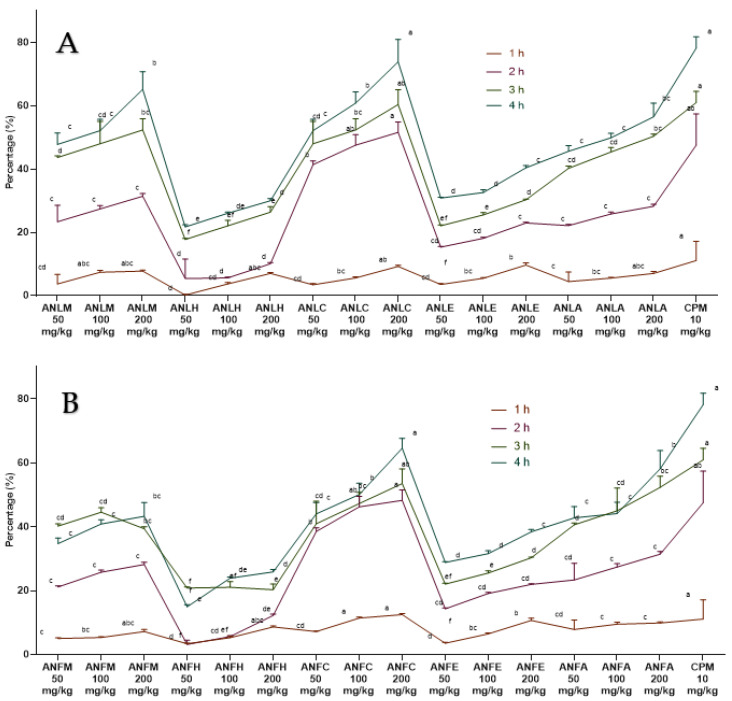
Percentage inhibition of *A. nitida* on histamine induced paw edema in rats (**A**) *A. nitida* leaves methanolic extract (ANLM), *A. nitida* leaves n-hexane fraction (ANLH), *A. nitida* leaves chloroform fraction (ANLC), *A. nitida* leaves ethyl acetate fraction (ANLE), *A. nitida* leaves aqueous fraction (ANLA), and (**B**) *A. nitida* fruit methanolic extract (ANFM), *A. nitida* fruit n-hexane fraction (ANFH), *A. nitida* fruit chloroform fraction (ANFC), *A. nitida* fruit ethyl acetate fraction (ANFE), *A. nitida* fruit aqueous fraction (ANFA). CPM: chlorpheniramine maleate. Data were analyzed using seven replicates as mean ± SD by two-way ANOVA followed by Bonferroni’s test. Different letters represent the significance level of *p* < 0.05 with respect to all other samples “a” with reference to positive control, “b” with reference to methanolic extract and “c, d, e, f” with reference to n-hexane, chloroform, ethyl acetate and aqueous fraction, respectively.

A moderate antihistaminic effect was detected for ANLC, ANFC (200 mg/kg) after 2 h of histamine administration and edema volume was remarkably (*p* < 0.01) repressed after 4 h with percent inhibition of 73.91 ± 7.09% and 64.56 ± 3.09% as compared to the standard drug chlorpheniramine maleate (78.26 ± 3.54%). The methanol extract and aqueous fractions of both organs also exhibited good antihistaminic activity (Figure 3 and Tables S5 and S6).

#### 2.2.4. Xylene-Induced Ear Edema

**Figure 4:**
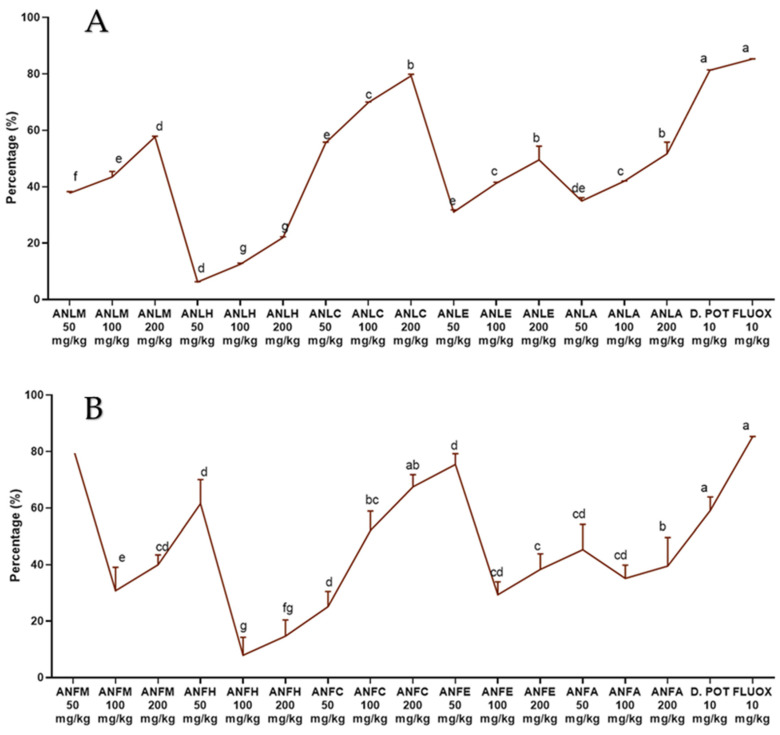
Percentage inhibition of *A. nitida* on xylene-induced ear edema in rats (**A**) *A. nitida* leaves methanolic extract (ANLM), *A. nitida* leaves n-hexane fraction (ANLH), *A. nitida* leaves chloroform fraction (ANLC), *A. nitida* leaves ethyl acetate fraction (ANLE), *A. nitida* leaves aqueous fraction (ANLA) and (**B**) *A. nitida* fruit methanolic extract (ANFM), *A. nitida* fruit n-hexane fraction (ANFH), *A. nitida* fruit chloroform fraction (ANFC), *A. nitida* fruit ethyl acetate fraction (ANFE), *A. nitida* fruit aqueous fraction (ANFA). D.POT: Diclofenac potassium; FLUOX: Fluoxetine. Data were analyzed using seven replicates as mean ± SD by one way ANOVA followed by Tukey’s test. Different letters represent the significance level of *p* < 0.05 with respect to all other samples “a, b” with reference to positive controls, “c” with reference to methanolic extract and “d, e, f, g” with reference to n-hexane, chloroform, ethyl acetate and aqueous fraction, respectively.

The results of leaves and fruits showing a dose- and time-dependent decline in edema are shown in Figure 4 and Tables S7 and S8. Saline rats exhibited an increase in ear weight (13.97 ± 0.01 mg), while the ANLC administration inhibited the gain in weight gain by 69.87 ± 0.19% (4.21 ± 0.02 mg) and 79.32 ± 0.58% (2.89 ± 0.08 mg) for 100 and 200 mg/kg, respectively. Likewise, ANFC remarkably (*p* < 0.01) inhibited a gain in weight by 75.42 ± 3.84% at 200 mg/kg dosage to the rats. The positive control diclofenac potassium and fluoxetine depicted 81.32 ± 0.15% (2.61 ± 0.02 mg) and 85.31 ± 0.04% (2.35 ± 0.04 mg) decrease in weight gain at 10 mg/kg, which was comparable to the doses of different fractions of *A. nitida*.

### 2.3. Analgesic Activity of A. nitida

#### 2.3.1. Hotplate Analgesic Test

**Figure 5:**
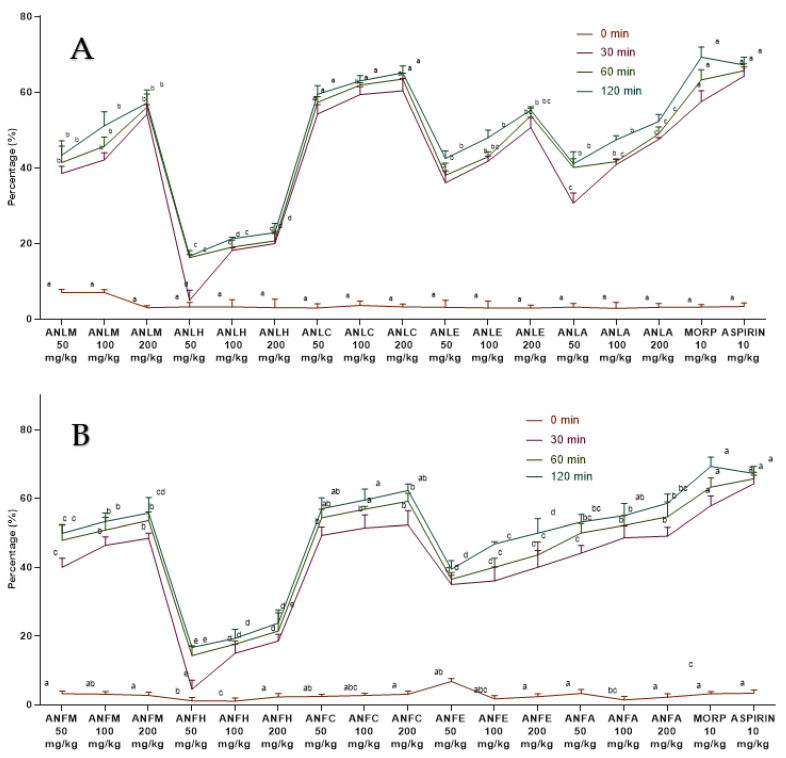
Percentage inhibition of *A. nitida* on hot plate test in rats (**A**) *A. nitida* leaves methanolic extract (ANLM), *A. nitida* leaves n-hexane fraction (ANLH), *A. nitida* leaves chloroform fraction (ANLC), *A. nitida* leaves ethyl acetate fraction (ANLE), *A. nitida* leaves aqueous fraction (ANLA) and (**B**) *A. nitida* fruit methanolic extract (ANFM), *A. nitida* fruit n-hexane fraction (ANFH), *A. nitida* fruit chloroform fraction (ANFC), *A. nitida* fruit ethyl acetate fraction (ANFE), *A. nitida* fruit aqueous fraction (ANFA). MORP: morphine. Data were analyzed using seven replicates as mean ± SD by two-way ANOVA followed by Bonferroni’s test. Different letters represent the significance level of *p* < 0.05 with respect to all other samples “a, b” with reference to positive controls, “c” with reference to methanolic extract and “d, e, f, g” with reference to n-hexane, chloroform, ethyl acetate and aqueous fraction, respectively.

The *A. nitida* leaves and fruits methanol extract and their fractions displayed a dose-dependent rise (Figure 5) in latency time and repressed pain perception in a pattern close to morphine and aspirin. The effect of ANLC was shown to be quite pronounced (*p* < 0.01) after 120 min at 200 mg/kg dosage relative to standard drug (Figure 5A and Table S9). In fruit, similar dose-dependent results were shown by ANFC with an inhibition of 62.31 ± 1.86% after 120 min of administration (Figure 5B and Table S10).

#### 2.3.2. Acetic Acid-Induced Writhing Test

**Figure 6:**
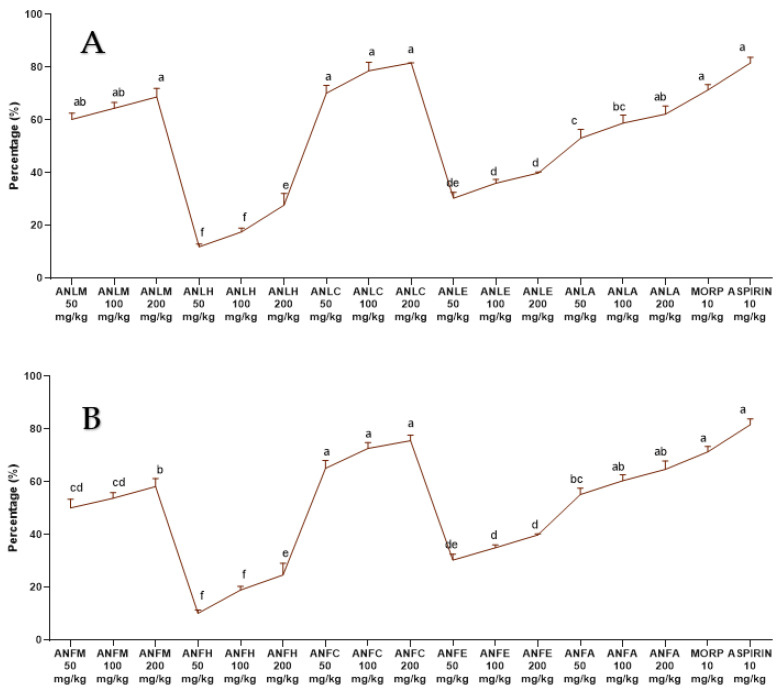
Percentage inhibition of *A. nitida* on acetic acid induced writhing in rats (**A**) *A. nitida* leaves methanolic extract (ANLM), *A. nitida* leaves n-hexane fraction (ANLH), *A. nitida* leaves chloroform fraction (ANLC), *A. nitida* leaves ethyl acetate fraction (ANLE), *A. nitida* leaves aqueous fraction (ANLA) and (**B**) *A. nitida* fruit methanolic extract (ANFM), *A. nitida* fruit n-hexane fraction (ANFH), *A. nitida* fruit chloroform fraction (ANFC), *A. nitida* fruit ethyl acetate fraction (ANFE), *A. nitida* fruit aqueous fraction (ANFA). MORP: morphine. Data were analyzed using seven replicates as mean ± SD by one way ANOVA followed by Tukey’s test. Different letters represent the significance level of *p* < 0.05 with respect to all other samples: “a, b” with reference to positive controls, “c” with reference to methanolic extract and “d, e, f, g” with reference to n-hexane, chloroform, ethyl acetate and aqueous fraction, respectively.

The ANLM and its resulting fractions ANLC and *A. nitida* leaves aqueous fraction (ANLA) considerably (*p* < 0.01) and dose-dependently (50, 100 and 200 mg/kg) decreased the number of abdominal contractions tempted after the acetic acid administration to the rats. The preventive effect of aspirin and morphine was non-considerably different from the defensive effect after exposure by ANLC and ANLM (50, 100 and 200 mg/kg) (Figure 6A and Table S11). Similar results were recorded for ANFC with 75.62 ± 2.06% inhibition of writhing (Figure 6B and Table S12). The writhing test in mice is regarded as being relatively reliable for the experimental evaluation of analgesic agents. However, using rats may have limitations as the challenge of the irritant can’t be repeated at short intervals and chronic challenges of irritants may cause injury to abdominal organs [28].

### 2.4. GC-MS Analysis

**Figure 7:**
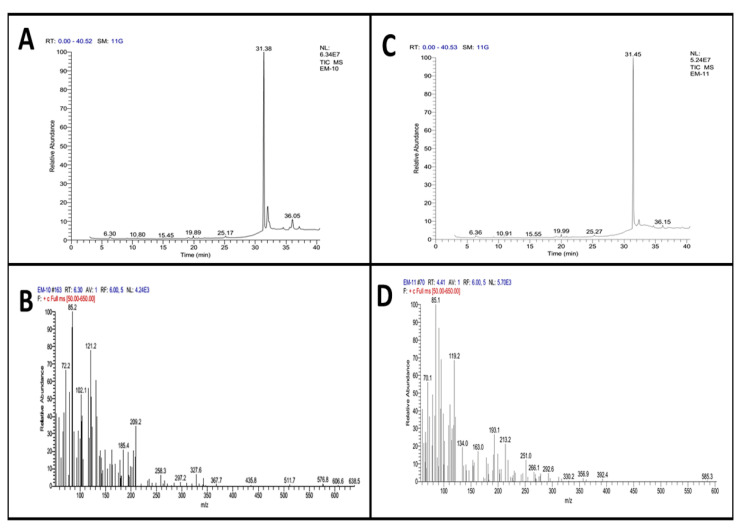
GC-MS graph presenting retention time and m/z ration (**A**) leaves retention time and (**C**) fruits retention time, (**B**) leaves m/z ration and (**D**) fruit m/z ration.

The methanolic extract of *A. nitida* leaves and fruit was subjected to GC-MS analysis to identify the bioactive compounds responsible for anti-inflammatory and analgesic effects. The graph of GC-MS exploration of ANLM (Figure 7A,B) and *A. nitida* fruit methanolic extract (ANFM) are illustrated (Figure 7C,D). Moreover, detail of 23 eluted components from ANLM and 24 from ANFM are presented in Table 1 and Table 2, respectively. The identification of bioactive compounds was based on a comparison of mass spectra and relative retention times with NST/NBS and Wiley spectral libraries (Table 1 and Table 2). In ANLC, total 23 compounds were identified, and their relative abundance is documented on the basis of peak area consisting of 1 alkenes (1.2%), 3 alcohols (7.25%), 1 amine (0.07%), 1 arene (0.33%), 1 terpene alcohols (2.59%), 2 ketones (2.05%), 5 esters (68.07%), 1 phosphol (15.23%), 1 acid amide (0.10%), 1 terpenoid (1.68%), 5 alkanes (0.67%) and 1 carotenoid (0.11%). In ANFC, there were 2 acid (0.12%), 2 alkenes (2.18%), 1 thioether (0.41%), 1 isothiazolinones (0.07%), 1 terpene (0.07%), 5 alcohols (8.62%), 1 terpene alcohols (2.61%), 5 esters (80.57%), 2 ketones (0.51%), 1 ether (0.33%), 1 benzopyridines (1.83%), 1 alkane (0.07) and 1 fatty acid (0.10%).

## 3. Discussion

Plants have been a standard reservoir of drugs for centuries, especially in developing countries. In fact, many presently used drugs originated either directly or indirectly from plant sources [29]. In current study, firstly, we investigated the toxicity of *A. nitida* leaves and fruit extracts/fractions in mice. The administration dose of 2000 mg/kg did not produce any sign of toxicity nor morality. Next, we investigate the anti-inflammatory activity of *A. nitida* leaves and fruit extracts/fractions, as carrageenan is broadly utilized to prompt inflammation in rats to validate anti-inflammatory action of herbs. Edema development is a triphasic phenomenon involving the production of inflammatory markers. Early phase of edema (0–1 h) is endorsed by the discharge of bradykinin, histamine and 5-hydroxytryptamine. The second and third phase (1–6 h) results in enhanced levels of cyclooxygenase and prostaglandins in rats [30]. Younis et al. [31] described the inhibition of NO with *Fraxinus xanthoxyloides* extracts in RAW-264.7 cells. The treatment of ANLC and ANFC exhibited anti-edematous consequences during early and late phases of edema development by hindering the discharge of inflammatory mediators. The occurrence of phytol might be accountable towards the anti-inflammatory potential by inhibiting neutrophil transfer, which is partially due to the suppression of IL-1β, TNF-α and oxidative stress levels [32].

Rheumatoid arthritis is a systemic inflammatory syndrome affecting about 1% population worldwide. Increased production of IL-1 and TNF-α plays a vital role in its pathogenesis. IL-1 and TNF-α increase the production of metalloproteinases and propagation of synovial cells, which results in the degradation of cartilage [33]. The declaration of several inflammatory mediators, such as interleukin family and-TNF-α, have been assessed in serum by *Trigonella foenum* seeds extract in adjuvant-induced arthritis in animal models [34]. This study demonstrated that ANLC and ANFC showed an appreciable reduction of inflammation in adjuvant-induced arthritis in rats, suggesting its role in the destruction of pro-inflammatory mediators.

Histamine production is deliberated as a part of the immune mechanism and is released under inflammation responses. Histamine plays a role during inflammation through edema, vasodilation and recruitment of eosinophils and enhanced vascular permeability. It also regulates the proliferation of T cells, leukocyte function and migration, B cell maturation and differentiation and discharge of lysosomal enzymes in neutrophils [35]. During histamine induced paw edema, NO synthase plays a vital role. Therefore, suppressing nitric oxide, inflammation can be decreased as it is directly involved in macromolecular extravasation prompted by histamine induced inflammation. So, using this approach, NO suppression by Bixa orellena extract has been validated in rats [36]. In the present study, ANLC and ANFC significantly inhibited inflammation induced by histamine, signifying that the anti-inflammatory activity detected is because of the stabilization of the mast cell membranes and the inhibition of nitric oxide synthesis.

Xylene-induced ear edema is also a well-established animal model to evaluate the anti-acute inflammatory effect of *A. nitida* leaves and fruit. Xylene provokes behavioral effects tempted by nociceptors. These nociceptors activate the inflammatory phase involving series of stimuli comprising inflammation of the peripheral tissues and central sensitization [37]. Our study indicates that *A. nitida* extracts act as anti-inflammatory agents as they reduce ear edema in rats. These findings are in coherence with previous findings [38].

The folkloric remedies from the plant kingdom signify the presence of biologically active compounds. In the present study, ANLM and ANFM were subjected to GC-MS analysis and 23 compounds from leaves were eluted between 6.30–40.05 min with RSI range 369–948 and 24 compounds from fruit were eluted between 6.36–40.05 min with RSI range 350–934, respectively. From the analysis, the therapeutically important bio-constituents were detected, which might be responsible for potential anti-inflammatory and analgesic effects. The GC-MS screening of the methanolic extract of leaves exhibited the existence of Neophytadiene, an alkene which contains significant anti-inflammatory properties and might be contributing to the anti-inflammatory potential of the ANLC fraction [39]. Likewise, the presence of 3,7,11,15-Tetramethyl-2-hexadecen-1-ol (terpene Alcohol), phenanthrene (arene) and phytol (diterpene alcohol), which are potential therapeutic anti-inflammatory agents, might contribute to the anti-inflammatory properties of ANLC and other fractions in crude extracts [32,40,41,42]. GC-MS screening of fruit has also shown the presence of neophytadiene, 3,7,11,15-Tetramethyl-2-hexadecen-1-ol (terpene alcohol) and Vitamin E (alcohol), which have been reported to exhibit anti-inflammatory effects, might be contributing towards the anti-inflammatory aptitude of ANFM [39,42].

The hot plate method (central type) and writhing test (peripheral type) are well-established methods in assessing analgesic potential of medicinal agents. In the hot plate method, ANLC and ANFC presented the best analgesic activity among all tested samples. Morphine excites analgesic effect by the initiation of opioid-receptors and the comparison of morphine and extracts represents the fact that both might cause similar effects to reduce pain sensations. In the writhing test, chloroform fraction of *A. nitida* (leaves and fruit) represented prominent analgesic activity, signifying that it has an analgesic effect on both the peripheral as well as on the central nervous systems. The presence of phytol, a diterpene alcohol and vitamin E in ANLM and ANFM revealed by GC-MS analysis, was reported to possess strong anti-nociceptive properties, reducing sensitivity to painful stimuli [32,43]. The compound phytol has been described in plant extracts, revealing antioxidant as well as anti-nociceptive effects, it was also reported to cause a substantial rise in latency in the hot plate test [44]. These anti-inflammatory and analgesic effects of extracts specifically ANLC and ANFC are encouraging, so the new medicines with improved biological actions and minimum side effects need to be developed.

## 4. Materials and Methods

### 4.1. Plant Sampling

The leaves and fruits of *A. nitida* were collected from Charbagh town (34.8358° N, 72.4436° E) in the district of Swat, Khyber Pakhtunkhwa province, Pakistan. The samples were identified by Dr. Mir Ajab Khan, Professor at the Department of Plant Sciences, and a specimen was submitted after authentication (127963) to the Herbarium of Pakistan Museum of Natural History, Islamabad, Pakistan.

### 4.2. Extract Preparation

The leaves and fruits of *A. nitida* were shade-dried at room temperature for two weeks and powdered in a Willy Mill to 80-mesh size. The fine powder (1 kg each) was macerated in 3L of commercial grade methanol (97%) for 10 days with frequent shaking. After maceration the extract was filtered with Whatman No.1 filter paper. The process was repeated thrice, and the filtrate obtained was mixed before evaporation with rotary evaporator under vacuum. A part of the leaves (ANLM) and fruits (ANFM) methanolic extracts were suspended in distilled water and fractionated by adding solvents in increasing polarity: n-hexane (ANLH and ANFH), chloroform (ANLC and ANFC), ethyl acetate (ANLE and ANFE) and residual (ANLA and ANFA) were used as an aqueous extract [45]. It is experimentally proven that extraction solvents play an important role in the extraction of important bioactive compounds from plant. n-hexane and chloroform tend to extract nonpolar compounds, while ethyl acetate has a partial polar and partial nonpolar extraction ability. On the other hand, aqueous extract consists of polar compounds. So, by utilizing this range of solvents we can extract polar, amphoteric and nonpolar compounds present in plants, which might be responsible for the diverse pharmacological activities.

### 4.3. Animals

Rats (Sprague-Dawley) of either sex, weighing (170–220 g) at 6 weeks old were kept at 25 ± 2 °C with a 12 h light/dark cycle at the Animal Facility of Quad-i-Azam University Islamabad, Pakistan (QAUIP). Rats were provided with healthy feed and laboratory conditions. The ethical approval for the use of rats for research and care (275-BCH) was acquired from the Ethics Committee of QAUIP. In each experiment, rats were divided into normal control, inflammation/pain-induced control, positive controls and experimental groups with seven rats in each treatment, and test samples were administered orally in each group.

### 4.4. Acute Toxicity Study

The acute toxicity of all the extract/fractions was investigated according to Guideline 425 of the Organization for Economic Co-operation and Development (OECD, 2001). For this, rats were treated at single dose of 2000 mg/kg, i.p., and saline (10 mL/kg) separately for each/fraction of both samples (leaves and fruits). The doses were prepared in normal saline and administered orally. The toxicity was examined after 24 h, following the behavior and clinical symptoms for 15 days.

### 4.5. Assessment of Anti-Inflammatory Activity

#### 4.5.1. Carrageenan-Induced Hind Paw Edema Test

Rats were divided into different groups comprising of 7 rats in each [46]. Group I was administered with 0.9% saline and the other groups were administered with samples (leaves/fruit) and two positive controls, including diclofenac potassium and fluoxetine at different concentrations. After 30 min, 0.1 mL (1%) of carrageenan was injected in the sub-plantar region of the right hind paw of each rat. The edema development was recorded with plethysmometer after 1, 2, 3 and 4 h of injection to rats and the percentage of inhibition was determined by the given formula. Data were analyzed using seven replicates by two-way ANOVA followed by Bonferroni’s test in GraphPad Prism with F value 102 for fruit and 99.6 for leaves, respectively.

$$\mathrm{Percent inhibition}=\left(\begin{array}{c} \underline{\mathrm{Edema in control rat}-\mathrm{Edema in test rat}}\\ \mathrm{Edema in control rat} \end{array}\right)\times 100$$

#### 4.5.2. Freunds’ Complete Adjuvant Induced Arthritis

To investigate the chronic inflammation Freunds’ complete adjuvant-induced arthritis model [47] was used. Rats were administered with extract/fractions as above while arthritis was induced with a 0.1 mL (1 mg) intradermal injection of *Mycobacterium tuberculosis* (heat-killed) into the sub-plantar region of the left hind paw of rats. Diclofenac potassium and fluoxetine served as positive controls. The inhibition of edema development was determined after 1, 2, 3 and 4 h, as previously described [47]. Data were analyzed using seven replicates by two-way ANOVA followed by Bonferroni’s test in GraphPad Prism with F value 106 for fruit and 105 for leaves, respectively.

#### 4.5.3. Histamine Induced Paw Edema Method

Previously reported histamine induction method was used to evaluate the antihistamine potential of the samples [48]. Chlorpheniramine maleate (antihistamine) served as the positive control drug. After 30 min of dosage, 0.1 mL (1 mg) of histamine was injected into the sub-plantar region of the left hind paw of each rat. Paw volume was measured using plethysmometer at 1, 2, 3 and 4 h of histamine injection and percent inhibition was measured as formerly described. Data were analyzed using seven replicates by two-way ANOVA followed by Bonferroni’s test in GraphPad Prism with F value 43.8 for fruit and 42.2 for leaves, respectively.

#### 4.5.4. Xylene-Induced Ear Edema Method

In this assay, edema inhibition was investigated as described previously [49]. Diclofenac potassium and fluoxetine served as positive controls. For edema development, 0.03 mL of xylene was applied on the left ear of each rat after 30 min of extract/fraction treatment. In this case, the left ear served as the control. After 15 min, the rats were euthanized using ketamine. Using a corkborer, 6 mm circular sections of both ears were taken, and the percent of inhibition was calculated. Data were analyzed using seven replicates by one way ANOVA followed by Tukey’s test in GraphPad Prism with F value 97 for fruit and 1271 for leaves, respectively.

### 4.6. Assessment of Analgesic Activity

#### 4.6.1. Hotplate Analgesic Test

The hot plate procedure was used to determine analgesic activity as described previously [46]. Animals exhibiting latency time greater than 15 s on a Harvard Apparatus set at 55 ± 2 °C were excluded in this experiment. The animals were grouped and treated with the extract/fraction as described previously along with positive control drugs (morphine and aspirin) [50,51]. After 30 min of dosage, the latency time was recorded for each rat on a hotplate maintained at 55 ± 2 °C, showing the number of lickings and jumpings. The cut-off time was set at 30 sec in order to avoid tissue damage. The latency time was recorded after 30, 60, 90 and 120 min and percent analgesia was measured by given formula. Data were analyzed using seven replicates by two-way ANOVA followed by Bonferroni’s test in GraphPad Prism with F value 61 for fruit and 104 for leaves, respectively.

$$\%\;\mathrm{Analgesia}=\left(\begin{array}{c} \underline{\mathrm{Test latency}-\mathrm{Control latency}}\\ \mathrm{Cut off time}-\mathrm{Control latency} \end{array}\right)\times 100$$

#### 4.6.2. Acetic Acid-Induced Writhing Test

The scheme used for this test was previously described by Khan et al. [52]. Rats were treated with extract/fractions or positive control drugs (morphine and aspirin) as given above. Acetic acid (1%) was administered after 30 min of treatment and the writhing movements were documented after 5 min of acetic acid induction over a period of 10 min [53]. Data were analyzed using seven replicates by one way ANOVA followed by Tukey’s test in GraphPad Prism with F value 583 for fruit and 622 for leaves, respectively. The percentage of inhibition was determined by the formula:

$$\mathrm{Percent inhibition}=\left(\begin{array}{c} \underline{\mathrm{Wriths in control rat}-\mathrm{Wriths in test rat}}\\ \mathrm{Wriths in control rat} \end{array}\right)\times 100$$

### 4.7. Gas Chromatography–Mass Spectrometry (GC-MS) Analysis

GC-MS analysis of ANLM and ANFM was performed on a “Thermo GC-Trace Ultra Ver; 5.0” gas chromatography attached with a “Thermo MS DSQ II” for mass evaluation. Components were detached on a “ZB 5-MS Capillary Standard Non-Polar Column” with 600 m length with a 0.25-μm film thickness. During the experiment, temperature was elevated from 70 to 260 °C at a rate of 6 °C/min. The flow-rate of carrier gas, helium, was 10 mL/min, whereas injection volume of the samples was 1 μL. The identification of the chemical components was performed on the basis of comparison of their respective retention time and mass spectra with those attained from authentic sample and/or NIST/NBS and Wiley spectral libraries [54] and determined the mass spectral match on the basis of the Match Factor (SI) or Reverse Match Factor (RSI) thresholds as reported by Gujar et al. [55].

### 4.8. Statistical Analysis

All data are expressed as the Mean ± SD with statistical differences (*p* < 0.05) between groups using one-way ANOVA following Tukey’s multiple comparison test while two-way ANOVA with Bonferroni’s multiple comparisons test was applied to the rest of the experimental models using GraphPad Prism version 8.1.0 software. Sample size and animal number for each experimental group was calculated using the ARRIVE guidelines [56].

## 5. Conclusions

The anti-inflammatory effect of *A. nitida* extract/fractions were evaluated in the present study. Results obtained show that the chloroform fraction of *A. nitida* leaves and fruit have significant anti-inflammatory and analgesic properties. The synergistic effects of nonpolar and other phytoconstituents might be responsible for the anti-inflammatory and analgesic activity of the leaves and fruits of *A. nitida*. Taken together, these findings provide evidence for the anti-inflammatory and analgesic activity of *A. nitida* and could explain the benefits of the traditional use of this plant.

## Figures and Tables

**Table 1:** GC-MS analysis of *A. nitida* leaves crude extract.

S. No.	Compound	Area %	Class	RT	SI	RSI
1.	Phytol	1.10	Diterpene alcohol	25.17	792	798
2.	Lucenin 2	1.95	Ketone	37.18	429	437
3.	Hexadecanoic acid, 2-phenyl-1,3-dioxan-5-yl ester	0.17	Ester	39.08	347	392
4.	5-Phenyl-4-trimethylsilyldibenzophosphole	15.23	Phosphol	32.02	726	916
5.	3Z-3′S-gamma-IRONE	0.31	Alkene	10.80	768	907
6.	2-(Phenylsulfanyl)cyclohexanol	0.76	Alcohol	39.64	391	681
7.	Bicyclo [3.2.0]hepta-2,6-diene	0.16	Deuterated cyclic Alkane	37.81	386	796
8.	2-tert-Butyl-4-trifluoromethyl-1-methylimidazole	0.07	Amine	12.96	866	948
9.	Rhodopin	0.11	Carotenoid	38.59	380	387
10.	Phenanthrene	0.33	Arene	21.73	389	727
11.	trans-4,7-Dimethyl-6,7-dihydro-5H-cyclopenta[c]pyridin-5-ol	0.09	Alcohol	10.30	503	761
12.	Cyclohexane, 1,1′,1″,1‴-(1,6-hexanediylidene)tetrakis	0.08	Alkane	36.57	413	427
13.	15-Isobutyl-(13àH)-isocopalane	0.32	Alkane	33.09	339	369
14.	3,7,11,15-Tetramethyl-2-hexadecen-1-ol	0.26	Terpene Alcohol	20.77	848	875
15.	1,2-Benzenedicarboxylic acid, mono(2-ethylhexyl) ester	65.38	Ester	31.38	606	754
16.	1,3,5-Trimethyl-2-octadecylcyclohexane	0.11	Alkane	33.58	419	434
17.	Neophytadiene	0.89	Alkene	19.89	867	887
18.	1,2-Benzenedicarboxylic acid, bis(2-ethylhexyl) ester	0.18	Ester	30.79	541	690
19.	Ethanone	0.10	Ketone	23.56	395	433
20.	Thiopheno[b,b′]dicamphore 1,1-dioxide	1.68	Terpenoid	35.62	552	862
21.	4-Bromobutanoic acid, tridec-2-ynyl ester	0.06	Ester	22.25	872	569
22.	Terephthalic acid, dodecyl 2-ethylhexyl ester	1.14	Ester	34.57	553	641
23.	6-Amino-1-[2-(3,4-dimethoxy-phenyl)-ethyl]-1H-pyrimidine-2,4-dione	0.10	Acid Amide	28.98	378	453

Where RT is the retention time, SI is the Match Factor (SI) and RSI is the Reverse Match Factor.

**Table 2:** GC-MS analysis of *A. nitida* fruit crude extract.

S. No.	Compound	Area %	Class	RT	SI	RSI
1.	Squalene	2.38	Triterpene	36.15	704	747
2.	Pluchidiol	1.08	Alcohol	19.20	400	565
3.	Neophytadiene	1.25	Alkene	19.99	858	881
4.	trans-8-Ethoxybicyclo [4.3.0]-3-nonene-7-carboxylic acid	0.07	Acid	4.41	422	425
5.	1-[(hexadeuterio)phenyl]naphthalene	0.93	Cyclic Alkene	39.30	395	745
6.	4-(p-Hydroxyphenyl)butyric acid	0.05	Acid	17.37	371	793
7.	3,7,11,15-Tetramethyl-2-hexadecen-1-ol	0.23	Terpene Alcohol	25.27	750	836
8.	2-Chloro-3-formylindol-1-carboxylic acid, 2,2,2-trichloroethyl ester	0.61	Ester	38.39	338	435
9.	5-(2-cyclohexen-1-ylthio)-1-phenyl-1H-tetrazole	2.30	Thioether	6.36	363	694
10.	15-methyltricyclo [6.5.2(13,14).0(7,15)]pentadeca-1,3,5,7,9,11,13-heptene	0.07	Terpene	13.09	806	862
11.	Olean-12-ene-3,15,16,21,22,28-hexol	1.43	Alcohol	37.01	334	350
12.	1,4-Benzenedicarboxylic acid, bis(2-ethylhexyl) ester	0.79	Ester	34.67	560	757
13.	24,25-Dihydroxycholecalciferol	0.12	Tri-Alcohol	36.71	395	455
14.	3Z-3′S-gamma-IRONE	0.40	Ketone	10.91	705	829
15.	tert-Butyl (Z)-3-(trifluoromethyl)-2-decenoate	0.10	Derivatives of fatty acids andorganoflurines	31.07	364	815
16.	1,2-Benzisothiazol-3(2H)-one, 2-(phenylmethyl)-, 1,1-dioxide	0.07	Isothiazolinones	29.10	359	665
17.	1-Cyclohexene-1-acrylic acid,2,6,6-trimethyl-3-oxo-,methyl ester	0.13	Ester	10.40	436	453
18.	4-(3-cyclohexylpropyl)-1,7-heptanediyl]bis	0.07	Alkane	27.04	384	407
19.	endo-(4aRS,8RS,8aRS)-8a-(Benzyloxycarbonyl)-1-oxo-2-(p-toluenesulfonyl)-8-(trimethylsiloxy)-1,2,3,4,4a,5,8,8a-octahydroisoquinoline	1.83	Benzopyridines	33.16	393	797
20.	Vitamin E	4.26	Alcohol	32.37	746	934
21.	1,2-Benzenedicarboxylic acid, mono(2-ethylhexyl) ester	77.87	Ester	31.45	502	619
22.	1-Methyl-4-(9H-xanthen-9-ylidene)piperidine	0.33	Ether	21.83	408	613
23.	Decahydronaphtho [2,3-b]furan-2-one, 3-[[(furan-2-ylmethyl)amino]methyl]-4a-hydroxy-5-methoxy-5,8a-dimethyl	0.69	Ester	30.21	349	432
24.	1-[4,4-Dimethyl-6-(2-oxopropyl)-1-oxaspiro [2.3]hex-2-yl]propan-2-one	0.11	Di-ketone	23.66	440	502

Where RT is the retention time, SI is the Match Factor (SI) and RSI is the Reverse Match Factor.

## Data Availability

Not applicable.

## Supplementary Materials

The following supporting information can be downloaded at: https://www.mdpi.com/article/10.3390/molecules27144582/s1, Table S1. Effect of *A. nitida* leaves extract and its fractions on carrageenan induced paw edema in rats; Table S2. Effect of *A. nitida* fruit extract and its fractions on carrageenan induced paw edema in rats; Table S3. Effect of *A. nitida* leaves extract and its fractions on Freunds’ complete adjuvant induced arthritis; Table S4. Effect of *A. nitida* fruit extract and its fractions on Freunds’ complete adjuvant induced arthritis; Table S5. Effect of *A. nitida* leaves extract and its fractions on histamine induced paw edema in rats; Table S6. Effect of *A. nitida* fruit extract and its fractions on histamine induced paw edema in rats; Table S7. Effect of *A. nitida* leaves extract and its fractions on xylene induced ear edema in rats; Table S8. Effect of *A. nitida* fruit and extract its fractions on xylene induced ear edema in rats; Table S9. Effect of *A. nitida* leaves extract and its fractions on hot plate test in rats; Table S10. Effect of *A. nitida* fruit extract and its fractions on hot plate test in rats; Table S11. Effect of *A. nitida* leaves extract and its fractions on acetic acid induced writhing in rats; Table S12. Effect of *A. nitida* fruit extract and its fractions on acetic acid induced writhing in rats.

## Sample Availability

Samples of the compounds are available from the authors.
