# Supplementary material for: Evaluation of Phytochemistry and Pharmacological Properties of Alnus nitida
Source: Molecules. 2022 Jul 18;27(14):4582. doi: 10.3390/molecules27144582 (PMC9320741; doi:10.3390/molecules27144582)
Supplement: Supplementary file 1 [file molecules-27-04582-s001.zip › molecules-1740704-supplementary.pdf]

Supplementary Materials

Evaluation of Phytochemistry and Pharmacological Properties of *Alnus nitida*

Table S1. Effect of *A. nitida* leaves extract and its fractions on carrageenan induced paw edema in rats

**Treatment**	**Dose/route**	**Before Injection**	**Increase in paw volume (ml) after carrageenan injection (mean ± SD)/ Percent inhibition of edema**			
			**+1 h**	**+2 h**	**+3 h**	**+4 h**
**Saline**	2 ml, i.p	1.14±0.13	2.42±0.27	2.36±0.48	1.98±0.37	1.64±0.28
**ANLM**	50 mg/kg, p.o	1.09±0.09	2.56±0.11 (2.45±0.92^{e})	2.07±0.02 (34.24±1.93^{c})	1.91±0.04 (48.66±2.38^{de})	1.36±0.10 (61.81±1.67^{d})
	100 mg/kg, p.o	1.06±0.09	2.52±0.10 (2.62±0.70^{e})	2.00±0.07 (36.59±3.44^{b})	1.84±0.05 (50.8±3.83^{d})	1.30±0.08 (64.75±3.04^{c})
	200 mg/kg, p.o	1.04±0.06	2.48±0.05 (4.19±1.24^{bcd})	1.94±0.12 (39.82±4.84^{b})	1.65±0.03 (61.47±2.51^{c})	1.13±0.06 (87.23± 1.52^{ab})
**ANLH**	50 mg/kg, p.o	1.18±0.05	2.65±0.07 (1.88±0.54^{d})	2.51±0.05 (9.87±1.78^{f})	2.49±0.03 (16.10±1.64^{g})	1.71±0.03 (22.11±2.05^{g})
	100 mg/kg, p.o	1.12±0.02	2.60±0.02 (1.78±0.26^{de})	2.44±0.02 (11.34±2.51^{f})	2.41±0.02 (16.20±2.74^{g})	1.63±0.01 (25.47±2.59^{g})
	200 mg/kg, p.o	1.11±0.05	2.59±0.06 (1.68±0.82^{e})	2.33±0.09 (19.10±2.03^{ef})	2.31±0.20 (27.44±4.53^{f})	1.43±0.07 (56.01±2.16^{d})
**ANLC**	50 mg/kg, p.o	1.13±0.04	2.61±0.07 (1.78±0.40^{e})	2.19±0.01 (29.44±2.96^{d})	1.81±0.02 (57.48±2.87^{cd})	1.37±0.04 (65.39±2.89^{cd})
	100 mg/kg, p.o	1.12±0.08	2.58±0.08 (2.84±0.96^{cd})	2.12±0.07 (33.36±2.88^{bc})	1.73±0.09 (59.01±3.52^{c})	1.34±0.08 (68.96±3.38^{c})
	200 mg/kg, p.o	1.08±0.05	2.51±0.06 (5.16±1.82^{bc})	1.70±0.03 (58.99±3.51^{a})	1.52±0.06 (73.43±2.45^{b})	1.15±0.05 (91.23±2.80^{a})
**ANLE**	50 mg/kg, p.o	1.08±0.08	2.56±0.08 (2.07±0.27^{d})	2.32±0.01 (16.72±2.06^{f})	2.24±0.06 (26.68±2.92^{f})	1.56±0.06 (30.30±3.33^{f})

	100 mg/kg, p.o	1.10±0.08	2.54±0.08 (4.10±1.32[bcd])	2.25±0.08 (22.50±2.16[de])	2.23±0.06 28.07±2.74[ef])	1.54±0.08 (36.39±2.36[f])
	200 mg/kg, p.o	1.10±0.07	2.56±0.07 (3.32±1.15[bcd])	2.20±0.10 (26.12±2.91[cde])	2.18±0.05 (31.41±4.97[e])	1.48±0.07 (45.01±2.87[e])
**ANLA**	50 mg/kg, p.o	1.09±2.56	2.56±0.07 (1.97±0.51[e])	2.11±0.05 (31.99±3.06[c])	1.92±0.04 (47.83±2.85[e])	1.43±0.05 (51.31±3.88[cd])
	100 mg/kg, p.o	1.09±0.07	2.56±0.07 (2.07±0.47[de])	2.07±0.07 (34.53±2.26[bc])	1.87±0.06 (50.98±2.69[d])	1.34±0.08 (63.71±1.98[c])
	200 mg/kg, p.o	1.10±0.08	2.57±0.07 (2.36±0.64[de])	1.99±0.11 (40.70±1.93[b])	1.76±0.13 (58.68±3.23[c])	1.23±0.08 (81.56±3.60[b])
**Diclofenac potassium**	10 mg/kg,i.p	1.10±0.09	2.49±0.08 (8.15±1.73[a)])	1.64±0.05 (64.63±3.82[a])	1.43±0.09 (79.97±2.47[a])	1.20±0.08 (86.63±3.42[ab)])
**Fluoxetine**	10 mg/kg,i.p	1.09±0.05	2.51±0.03 (5.54±1.74[b])	1.64±0.05 (63.50±2.68[a])	1.45±0.04 (77.70±2.62[ab])	1.20±0.06 (84.71±2.46[b])

ANLM; *A. nitida* leaves methanol extract, ANLH; *A. nitida* leaves n-hexane fraction, ANLC; *A. nitida* leaves chloroform fraction, ANLE; *A. nitida* leaves ethyl acetate fraction, ANLA; *A. nitida* leaves aqueous fraction. Values are presented as mean±SD (n=7). Means with different superscript [(a-g)] letters in column are significantly ($P < 0.01$) different from each other. Percentage inhibition is shown in brackets. Inhibition in saline treated group at each time point was calculated relative to paw edema after 1 h.

Table S2. Effect of *A. nitida* fruit extract and its fractions on Carrageenan induced paw edema in rats

Treatment	Dose/route	Before injection	Increase in paw volume (ml) after carrageenan injection (mean ± SD)/ Percent inhibition of edema			
			+1 h	+2 h	+3 h	+4 h
**Saline**	2 ml, i.p	1.14±0.13	2.42±0.27	2.36±0.48	1.98±0.37	1.64±0.28
**ANFM**	50 mg/kg, p.o	1.06±0.09	2.53±0.10 (2.18±0.20[f])	2.04±0.08 (34.34±1.93[d])	1.90±0.05 (46.72±1.79[e])	1.36±0.09 (55.51±2.64[de])
	100 mg/kg, p.o	1.06±0.10	2.52±0.10 (2.36±0.93[e])	2.00±0.08 (36.88±3.39[cde])	1.85±0.05 (49.96±3.24[d])	1.34±0.11 (58.87±3.38[d])
	200 mg/kg, p.o	1.04±0.06	2.48±0.06 (3.71±0.74[cde])	1.96±0.06 (38.45±2.71[cd])	1.67±0.04 (60.17±2.81[c])	1.19±0.05 (78.41± 3.92[c])
**ANFH**	50 mg/kg, p.o	1.10±0.08	2.58±0.08 (2.94±0.82[e])	2.30±0.08 (19.66±1.18[g])	2.29±0.08 (23.90±2.18[g])	1.58±0.07 (30.72±1.67[f])
	100 mg/kg, p.o	1.10±0.08	2.56±0.08 (3.13±0.77[de])	2.27±0.07 (21.62±1.89[fg])	2.27±0.07 (25.94±2.08[f])	1.56±0.07 (33.45±2.65[f])
	200 mg/kg, p.o	1.11±0.07	2.56±0.07 (3.52±1.29[cde])	2.20±0.10 (26.31±2.67[f])	2.16±0.05 (32.99±3.77[e])	1.45±0.06 (50.26±2.36[e])
**ANFC**	50 mg/kg, p.o	1.12±0.08	2.59±0.08 (2.46±0.94[ef])	2.17±0.07 (29.74±1.10[ef])	1.83±0.07 (55.71±3.16[d])	1.40±0.08 (59.50±3.29[e])
	100 mg/kg, p.o	1.12±0.08	2.58±0.08 (2.74±0.91[e])	2.12±0.08 (32.77±2.30[e])	1.79±0.08 (59.01±3.52[c])	1.37±0.07 (63.92±2.55[d])
	200 mg/kg, p.o	1.10±0.05	2.51±0.06 (6.51±0.94[ab])	1.71±0.03 (59.97±2.59[b])	1.53±0.06 (73.71±1.27[b])	1.19±0.06 (87.86±2.78[a])
**ANFE**	50 mg/kg, p.o	1.13±.0.02	2.60±0.02 (1.68±0.70[f])	2.44±0.03 (11.73±1.41[h])	2.40±0.02 (19.07±0.49[f])	1.59±0.03 (31.98±2.16[f])
	100 mg/kg, p.o	1.13±0.05	2.60±0.02 (2.07±0.85[e])	2.42±0.03 (12.81±1.53[h])	2.38±0.05 (20.37±1.63[f])	1.58±0.03 (33.45±2.46[f])
	200 mg/kg, p.o	1.11±0.05	2.59±0.06 (1.88±0.85[e])	2.29±0.09 (21.84±2.29[g])	2.31±0.26 (27.60±4.87[f])	1.48±0.08 (49.03±2.01[e])
**ANFA**	50 mg/kg, p.o	1.09±0.07	2.57±0.07 (1.88±0.66[f])	2.10±0.06 (32.18±1.79[e])	1.91±0.07 (48.76±1.79[de])	1.39±0.06 (56.98±2.36[e])

	100 mg/kg, p.o	1.09±0.07	2.56±0.07 (2.07±0.47[e])	2.07±0.07 (34.24±2.11[de])	1.88±0.09 (50.15±1.47[d])	1.36±0.08 (60.76±2.31[d])
	200 mg/kg, p.o	1.10±0.08	2.53±0.08 (4.96±1.06[bcd])	1.98±0.10 (41.58±1.18[c])	1.72±0.10 (61.47±4.68[c])	1.24±0.07 (80.93±3.38[bc])
**Diclofenac potassium**	10 mg/kg,i.p	1.10±0.09	2.49±0.08 (8.15±1.73[a])	1.64±0.05 (64.63±3.82[a])	1.43±0.09 (79.97±2.47[a])	1.20±0.08 (86.63±3.42[a])
**Fluoxetine**	10 mg/kg,i.p	1.09±0.05	2.51±0.03 (5.54±1.74[bc])	1.64±0.05 (63.50±2.68[ab])	1.45±0.04 (77.70±2.62[ab])	1.20±0.06 (84.71±2.46[ab])

ANFM; *A. nitida* fruit methanol extract, ANFH; *A. nitida* fruit n-hexane fraction, ANFC; *A. nitida* fruit chloroform fraction, ANFE; *A. nitida* fruit ethyl acetate fraction, ANFA; *A. nitida* fruit aqueous fraction. Values are presented as mean±SD (n=7). Means with different superscript [(a-d)] letters in column are significantly ($P < 0.01$) different from each other. Percentage inhibition is shown in brackets. Inhibition in saline treated group at each time point was calculated relative to paw edema after 1 h.

Table S3. Effect of *A. nitida* leaves extract and its fractions on Freunds' complete adjuvant induced arthritis

Treatment	Dose/route	Increase in paw volume (ml) after carrageenan injection (mean ± SD)/ Percent inhibition of edema			
		0 day	+7 day	+14 day	+21 day
**Saline**	2 ml, i.p	1.15±0.02	2.56±0.02	2.62±0.25	2.56±0.02
**ANLM**	50 mg/kg, p.o	1.14±0.02	2.31±0.02 (9.76±0.63^{cde})	1.89±0.01 (27.25±0.46^{e})	1.21±0.07 (52.45±2.78^{d})
	100 mg/kg, p.o	1.13±0.03	2.27±0.06 (11.32±2.31^{bcd})	1.76±0.04 (32.25±1.66^{d})	1.02±0.05 (60.04±1.89^{c})
	200 mg/kg, p.o	1.12±0.02	2.12±0.06 (17.07±2.56^{a})	1.56±0.07 (39.94±2.78^{c})	0.89±0.06 (65.12±2.47^{bc})
**ANLH**	50 mg/kg, p.o	1.15±0.04	2.45±0.04 (4.12±1.85^{g})	2.04±0.03 (21.37±1.27^{f})	1.83±0.04 (28.23±1.72^{e})
	100 mg/kg, p.o	1.13±0.01	2.41±0.03 (5.69±1.00^{fg})	1.99±0.03 (23.35±1.21^{f})	1.78±0.02 (30.35±0.80^{e})
	200 mg/kg, p.o	1.11±0.01	2.36±0.06 (7.47±2.26^{efg})	1.89±0.05 (27.25±2.22^{e})	1.67±0.03 (34.59±1.25^{e})
**ANLC**	50 mg/kg, p.o	1.09±0.01	2.33±0.03 (8.98±1.54d^{ef})	1.39±0.04 (46.53±1.64^{b})	0.91±0.12 (64.34±5.03^{bc})
	100 mg/kg, p.o	1.04±0.02	2.31±0.02 (9.70±0.91^{cde})	1.32±0.03 (49.12±1.48^{b})	0.80±0.08 (68.69±3.19^{b})
	200 mg/kg, p.o	1.02±0.02	2.23±0.07 (12.89±2.95^{bc})	1.22±0.06 (52.96±2.14^{a})	0.62±0.01 (75.44±6.65^{a})
**ANLE**	50 mg/kg, p.o	1.11±0.01	2.41±0.02 (5.85±0.63^{f})	1.99±0.02 (23.30±0.69^{d})	1.78±0.01 (30.46±0.31^{e})
	100 mg/kg, p.o	1.12±0.02	2.37±0.02 (7.42±0.63^{fg})	1.84±0.04 (29.23±1.67^{cd})	1.72±0.03 (32.81±1.27^{c})
	200 mg/kg, p.o	1.10±0.01	2.26±0.02 (11.71±0.63^{c})	1.72±0.03 (33.62±1.38^{b})	1.53±0.06 (40.23±2.65^{b})
**ANLA**	50 mg/kg, p.o	1.21±0.01	2.39±0.03	1.93±0.03	1.72±0.02

			(6.41±1.14[g])	(25.71±1.20[cd])	(32.58±0.80[c])
	100 mg/kg, p.o	1.14±0.02	2.31±0.05 (9.59±2.04[de])	1.79±0.01 (31.15±0.31[bc])	1.68±0.11 (34.31±4.45[c])
	200 mg/kg, p.o	1.09±0.01	2.21±0.04 (13.61±1.69[b])	1.67±0.02 (35.71±0.84[b])	1.45±0.02 (43.35±1.14[b])
**Diclofenac potassium**	10 mg/kg,i.p	1.11±0.15	2.21±0.02 (13.56±0.86[ab])	1.23±0.03 (52.69±0.96[a])	0.62±0.02 (75.78±0.92[a])
**Fluoxetine**	10 mg/kg,i.p	1.13±0.03	2.19±0.04 (15.22±1.21[a])	1.22±0.01 (51.11±2.13[a])	0.59±0.07 (74.32±2.31[a])

ANLM; *A. nitida* leaves methanol extract, ANLH; *A. nitida* leaves n-hexane fraction, ANLC; *A. nitida* leaves chloroform fraction, ANLE; *A. nitida* leaves ethyl acetate fraction, ANLA; *A. nitida* leaves aqueous fraction. Values are presented as mean±SD (n=7). Means with different superscript (a-g) letters in column are significantly ($P < 0.01$) different from each other. Percentage inhibition is shown in brackets.

Table S4. Effect of *A. nitida* fruit and its fractions on Freunds' complete adjuvant induced arthritis

Treatment	Dose/route	Increase in paw volume (ml) after carrageenan injection (mean ± SD)/ Percent inhibition of edema			
		0 day	+7 day	+14 day	+21 day
**Saline**	2 ml, i.p	1.15±0.02	2.56±0.02	2.62±0.25	2.56±0.02
**ANFM**	50 mg/kg, p.o	1.14±0.02	2.39±0.03 (6.42±1.14[g])	1.93±0.03 (24.71±1.20[cd])	1.72±0.02 (32.58±0.80[c])
	100 mg/kg, p.o	1.13±0.03	2.31±0.05 (9.59±2.04[de])	1.79±0.01 (31.15±0.31[bc])	1.68±0.11 (34.31±4.45[c])
	200 mg/kg, p.o	1.12±0.02	2.21±0.04 (13.61±1.69[b])	1.66±0.02 (36.71±0.84[b])	1.43±0.02 (44.35±1.14[b])
**ANFH**	50 mg/kg, p.o	1.15±0.04	2.41±0.04 (4.52±1.85[g])	2.04±0.03 (21.37±1.27[f])	1.89±0.04 (25.23±1.72[e])
	100 mg/kg, p.o	1.13±0.01	2.34±0.03 (6.69±1.00[fg])	1.99±0.03 (23.35±1.21[f])	2.18±0.02 (26.35±0.80[e])
	200 mg/kg, p.o	1.11±0.01	2.36±0.06 (7.47±2.26[efg])	1.89±0.05 (27.25±2.22[e])	2.29±0.03 (31.59±1.25[e])
**ANFC**	50 mg/kg, p.o	1.09±0.01	2.35±0.03 (7.98±1.54d[ef])	1.39±0.04 (46.53±1.64[b])	1.21±0.12 (60.34±5.03[bc])
	100 mg/kg, p.o	1.04±0.02	2.31±0.02 (9.70±0.91[cde])	1.32±0.03 (49.12±1.48[b])	1.10±0.08 (65.69±3.19[b])
	200 mg/kg, p.o	1.02±0.02	2.14±0.07 (15.89±2.95[a])	1.22±0.06 (52.96±2.14[a])	0.98±0.01 (70.44±6.65[a])
**ANFE**	50 mg/kg, p.o	1.11±0.01	2.40±0.02 (6.85±0.63[f])	1.99±0.02 (23.30±0.69[d])	1.98±0.01 (28.46±0.31[e])
	100 mg/kg, p.o	1.12±0.02	2.37±0.02 (7.42±0.63[fg])	1.88±0.04 (29.23±1.67[cd])	1.79±0.03 (31.81±1.27[c])
	200 mg/kg, p.o	1.10±0.01	2.26±0.02 (11.71±0.63[c])	1.76±0.03 (31.62±1.38[b])	1.73±0.06 (39.23±2.65[b])
**ANFA**	50 mg/kg, p.o	1.21±0.01	2.31±0.02 (9.76±0.63[cde])	1.89±0.01 (27.25±0.46[e])	1.21±0.07 (52.45±2.78[d])

	100 mg/kg, p.o	1.14±0.02	2.37±0.06<br>(10.32±2.31^{bcd})	1.75±0.04<br>(33.25±1.66^{d})	1.08±0.05<br>(59.04±1.89^{c})
	200 mg/kg, p.o	1.09±0.01	2.02±0.06<br>(18.07±2.56^{a})	1.59±0.07<br>(38.94±1.78^{c})	0.99±0.06<br>(63.12±2.47^{bc})
**Diclofenac potassium**	10 mg/kg,i.p	1.11±0.15	2.21±0.02<br>(13.56±0.86^{ab})	1.23±0.03<br>(52.69±0.96^{a})	0.62±0.02<br>(75.78±0.92^{a})
**Fluoxetine**	10 mg/kg,i.p	1.13±0.03	2.19±0.04<br>(15.22±1.21^{a})	1.22±0.01<br>(51.11±2.13^{a})	0.59±0.07<br>(74.32±2.31^{a})

ANFM; *A. nitida* fruit methanol extract, ANFH; *A. nitida* fruit n-hexane fraction, ANFC; *A. nitida* fruit chloroform fraction, ANFE; *A. nitida* fruit ethyl acetate fraction, ANFA; *A. nitida* fruit aqueous fraction. Values are presented as mean±SD (n=7). Means with different superscript $^{(a-d)}$ letters in column are significantly ($P < 0.01$) different from each other. Percentage inhibition is shown in brackets.

Table S5. Effect of *A. nitida* leaves and its fractions on histamine induced paw edema in rats

Treatment	Dose/route	Increase in paw volume (ml) after histamine injection (mean ± SD)/ Percent inhibition of edema			
		+1 h	+2 h	+3 h	+4 h
**Histamine**	1 mg/ ml, i.p	0.27±0.01	0.248±0.02	0.231±0.03	0.23±0.04
**ANLM**	50 mg/kg, p.o	0.26±0.01 (3.70±3.02[cd])	0.19±0.01 (23.38±5.20[c])	0.13±0.01 (43.66±0.52[d])	0.12±0.08 (47.82±3.54[c])
	100 mg/kg, p.o	0.25±0.02 (7.40±0.60[abc])	0.18±0.02 (27.41±1.06[c])	0.12±0.01 (48.05±7.06[cd])	0.11±0.08 (52.17±3.54[c])
	200 mg/kg, p.o	0.24±0.01 (7.77±0.30[abc])	0.17±0.02 (31.45±0.83[c])	0.11±0.08 (52.38±3.53[bc])	0.08±0.01 (65.21±5.61[b])
**ANLH**	50 mg/kg, p.o	0.26±0.01 (0.21±0.19[d])	0.23±0.01 (5.41±1.31[d])	0.19±0.001 (17.74±0.35[f])	0.18±0.01 (21.73±0.66[e])
	100 mg/kg, p.o	0.26±0.01 (3.70±0.42[cd])	0.23±0.01 (5.64±0.23[d])	0.18±0.044 (22.07±1.76[ef])	0.17±0.01 (26.08±0.35[de])
	200 mg/kg, p.o	0.251±0.01 (7.03±0.30[abc])	0.22±0.01 (10.08±0.40[d])	0.17±0.04 (26.40±1.76[e])	0.16±0.01 (30.00±0.71[d])
**ANLC**	50 mg/kg, p.o	0.26±0.01 (3.43±0.41[cd])	0.14±0.02 (41.41±1.18[b])	0.12±0.01 (48.05±7.06[cd])	0.11±0.08 (52.17±3.54[c])
	100 mg/kg, p.o	0.25±0.01 (5.60±0.33[bc])	0.13±0.08 (47.58±3.29[ab])	0.11±0.02 (52.38±3.53[bc])	0.09±0.08 (60.86±3.54[b])
	200 mg/kg, p.o	0.24±0.01 (9.25±0.37[ab])	0.12±0.03 (51.61±3.29[a])	0.09±0.02 (60.42±4.62[ab])	0.06±0.01 (73.91±7.09[a])
**ANLE**	50 mg/kg, p.o	0.26±0.01 (3.54±0.29[cd])	0.21±0.03 (15.32±0.23[cd])	0.18±0.05 (22.07±0.24[ef])	0.15±0.01 (30.86±0.25[d])
	100 mg/kg, p.o	0.25±0.01 (5.50±0.25[bc])	0.20±0.08 (18.14±0.32[cd])	0.17±0.01 (25.54±0.70[e])	0.15±0.02 (32.60±0.90[d])
	200 mg/kg, p.o	0.24±0.01 (9.68±0.69[b])	0.19±0.05 (22.98±0.23[c])	0.16±0.02 (30.30±0.24[d])	0.13±0.01 (40.43±0.71[c])

**ANLA**	50 mg/kg, p.o	0.25±0.08 (4.44±0.20^{c})	0.19±0.03 (22.17±0.32^{c})	0.13±0.04 (40.25±0.70^{cd})	0.12±0.04 (45.65±1.77^{c})
	100 mg/kg, p.o	0.25±0.05 (5.55±0.21^{bc})	0.18±0.01 (25.80±0.61^{c})	0.12±0.03 (45.45±1.41^{cd})	0.11±0.03 (49.93±1.36^{c})
	200 mg/kg, p.o	0.25±0.01 (7.03±0.64^{abc})	0.17±0.01 (28.22±0.65^{c})	0.11±0.03 (50.40±0.65^{bc})	0.10±0.01 (56.52±4.34^{bc})
**Chlorpheniramine maleate**	25 mg/kg,i.p	0.24±0.02 (11.11±6.04^{a})	0.13±0.02 (47.58±9.87^{ab})	0.09±0.01 (61.03±3.53^{a})	0.05±0.01 (78.26±3.54^{a})

ANLM; *A. nitida* leaves methanol extract, ANLH; *A. nitida* leaves n-hexane fraction, ANLC; *A. nitida* leaves chloroform fraction, ANLE; *A. nitida* leaves ethyl acetate fraction, ANLA; *A. nitida* leaves aqueous fraction. Values are presented as mean±SD (n=7). Means with different superscript $^{(a\text{-}g)}$ letters in column are significantly (P < 0.01) different from each other. Percentage inhibition is shown in brackets.

Table S6. Effect of *A. nitida* fruit extract and its fractions on histamine induced paw edema in rats

Treatment	Dose/route	Increase in paw volume (ml) after histamine injection (mean ± SD)/ Percent inhibition of edema			
		**+1 h**	**+2 h**	**+3 h**	**+4 h**
**Histamine**	1 mg/ ml, i.p	0.27±0.01	0.248±0.02	0.231±0.03	0.23±0.04
**ANFM**	50 mg/kg, p.o	0.25±0.08<br>(5.04±0.20^{c})	0.19±0.03<br>(21.17±0.32^{c})	0.14±0.04<br>(40.25±0.70^{cd})	0.21±0.04<br>(34.75±1.77^{c})
	100 mg/kg, p.o	0.25±0.05<br>(5.34±0.21^{bc})	0.18±0.01<br>(25.80±0.61^{c})	0.13±0.03<br>(44.58±1.41^{cd})	0.17±0.03<br>(40.90±1.36^{c})
	200 mg/kg, p.o	0.25±0.01<br>(7.19±0.64^{abc})	0.18±0.01<br>(28.22±0.65^{c})	0.15±0.03<br>(39.40±0.65^{bc})	0.12±0.01<br>(43.31±4.34^{bc})
**ANFH**	50 mg/kg, p.o	0.26±0.03<br>(3.38±0.19^{d})	0.28±0.01<br>(3.14±1.31^{f})	0.18±0.001<br>(20.84±0.35^{f})	0.27±0.01<br>(14.96±0.66^{e})
	100 mg/kg, p.o	0.25±0.01<br>(5.29±0.42^{cd})	0.23±0.01<br>(5.64±0.23^{ef})	0.18±0.044<br>(21.07±1.76^{ef})	0.19±0.01<br>(23.95±0.35^{de})
	200 mg/kg, p.o	0.246±0.01<br>(8.67±0.30^{abc})	0.21±0.01<br>(12.26±0.40^{de})	0.26±0.04<br>(20.33±1.76^{e})	0.19±0.01<br>(25.91±0.71^{d})
**ANFC**	50 mg/kg, p.o	0.25±0.01<br>(7.19±0.41^{cd})	0.17±0.02<br>(38.55±1.18^{b})	0.19±0.01<br>(40.95±7.06^{cd})	0.16±0.08<br>(44.06±3.54^{c})
	100 mg/kg, p.o	0.24±0.01<br>(11.42±0.33^{a})	0.16±0.08<br>(46.27±3.29^{ab})	0.19±0.02<br>(47.28±3.53^{bc})	0.22±0.08<br>(50.14±3.54^{b})
	200 mg/kg, p.o	0.23±0.01<br>(12.48±0.37^{a})	0.14±0.03<br>(48.30±3.29^{a})	0.10±0.02<br>(53.56±4.62^{ab})	0.13±0.01<br>(64.56±3.09^{a})
**ANFE**	50 mg/kg, p.o	0.27±0.01<br>(3.54±0.29^{d})	0.22±0.03<br>(14.32±0.23^{cd})	0.18±0.05<br>(22.07±0.24^{ef})	0.18±0.01<br>(28.86±0.25^{d})
	100 mg/kg, p.o	0.25±0.01<br>(6.50±0.25^{bc})	0.19±0.08<br>(19.14±0.32^{cd})	0.17±0.01<br>(25.54±0.70^{e})	0.15±0.02<br>(31.60±0.90^{d})
	200 mg/kg, p.o	0.19±0.01<br>(10.68±0.69^{b})	0.20±0.05<br>(21.98±0.23^{cd})	0.16±0.02<br>(30.30±0.24^{d})	0.18±0.01<br>(38.43±0.71^{c})
**ANFA**	50 mg/kg, p.o	0.19±0.01<br>(7.82±3.02^{cd})	0.19±0.01<br>(23.38±5.20^{cd})	0.19±0.01<br>(40.66±0.52^{d})	0.17±0.08<br>(42.82±3.54^{c})

	100 mg/kg, p.o	0.24±0.02 (9.52±0.60^{c})	0.18±0.02 (27.41±1.06^{c})	0.15±0.01 (45.05±7.06^{cd})	0.22±0.08 (44.17±3.54^{c})
	200 mg/kg, p.o	0.25±0.01 (9.91±0.30^{c})	0.17±0.02 (31.45±0.83^{c})	0.11±0.08 (52.38±3.53^{bc})	0.19±0.01 (58.21±5.61^{b})
**Chlorphenira mine maleate**	25 mg/kg,i.p	0.24±0.02 (11.11±6.04^{a})	0.13±0.02 (47.58±9.87^{ab})	0.09±0.01 (61.03±3.53^{a})	0.05±0.01 (78.26±3.54^{a})

ANFM; *A. nitida* fruit methanol extract, ANFH; *A. nitida* fruit n-hexane fraction, ANFC; *A. nitida* fruit chloroform fraction, ANFE; *A. nitida* fruit ethyl acetate fraction, ANFA; *A. nitida* fruit aqueous fraction. Values are presented as mean±SD (n=7). Means with different superscript $^{(a-d)}$ letters in column are significantly ($P < 0.01$) different from each other. Percentage inhibition is shown in brackets.

Table S7. Effect of *A. nitida* leaves and its fractions on xylene induced ear edema in rats

Treatment	Dose/route	Mean increase in ear weight (mg) (mean ± SD)	Percent inhibition
**Saline**	2 ml, i.p	13.97±0.01	0
**ANLM**	50 mg/kg, p.o	8.70±0.08	37.70±0.58^{f}
	100 mg/kg, p.o	7.63±0.74	43.48±1.97^{e}
	200 mg/kg, p.o	5.91±0.03	57.66±0.22^{d}
**ANLH**	50 mg/kg, p.o	13.11±0.01	6.22±0.09^{h}
	100 mg/kg, p.o	12.23±0.05	12.51±0.36^{g}
	200 mg/kg, p.o	10.90±0.03	21.97±0.25^{g}
**ANLC**	50 mg/kg, p.o	6.20±0.03	55.58±0.25^{e}
	100 mg/kg, p.o	4.21±0.02	69.87±0.19^{c}
	200 mg/kg, p.o	2.89±0.08	79.32±0.58^{b}
**ANLE**	50 mg/kg, p.o	9.64±0.10	31.04±0.77^{e}
	100 mg/kg, p.o	8.21±0.05	41.22±0.38^{c}
	200 mg/kg, p.o	7.05±0.68	49.51±4.88^{b}
**ANLA**	50 mg/kg, p.o	9.09±0.16	34.92±1.19^{de}
	100 mg/kg, p.o	8.11±0.01	41.98±0.05^{c}
	200 mg/kg, p.o	6.75±0.57	51.68±4.13^{b}
**Diclofenac potassium**	10 mg/kg,i.p	2.61±0.02	81.32±0.15^{a}
**Fluoxetine**	10 mg/kg,i.p	2.35±0.04	85.31±0.04^{a}

ANLM; *A. nitida* leaves methanol extract, ANLH; *A. nitida* leaves n-hexane fraction, ANLC; *A. nitida* leaves chloroform fraction, ANLE; *A. nitida* leaves ethyl acetate fraction, ANLA; *A. nitida* leaves aqueous fraction. Values are presented as mean±SD (n=7). Means with different superscript $^{(a\text{-}g)}$ letters in column are significantly (P <0.01) different from each other.

Table S8. Effect of *A. nitida* fruit and its fractions on xylene induced ear edema in rats

Treatment	Dose/route	Mean increase in ear weight (mg) (mean ± SD)	Percent inhibition
**Saline**	2 ml, i.p	13.97±0.01	0
**ANFM**	50 mg/kg, p.o	9.68±1.15	30.73±8.28^{ef}
	100 mg/kg,	8.39±0.48	39.96±3.50^{e}
	200 mg/kg,	5.37±1.19	61.54±8.54^{cd}
**ANFH**	50 mg/kg, p.o	12.87±0.89	7.91±6.40^{h}
	100 mg/kg,	11.92±0.80	14.68±5.74^{gh}
	200 mg/kg,	10.46±0.75	25.12±5.37^{fg}
**ANFC**	50 mg/kg, p.o	6.69±0.96	52.08±6.89^{d}
	100 mg/kg,	4.54±0.59	67.52±4.28^{bc}
	200 mg/kg,	3.43±0.53	75.42±3.84^{ab}
**ANFE**	50 mg/kg, p.o	9.87±0.64	29.33±4.58^{d}
	100 mg/kg,	8.62±0.77	38.33±5.53^{cd}
	200 mg/kg,	7.64±1.25	45.30±8.95^{c}
**ANFA**	50 mg/kg, p.o	9.06±0.65	35.15±4.71^{cd}
	100 mg/kg,	8.45±1.41	39.49±10.10^{cd}
	200 mg/kg,	5.71±0.66	59.11±4.78^{b}
**Diclofenac**	10 mg/kg,i.p	2.61±0.02	81.32±0.15^{a}
**Fluoxetine**	10 mg/kg,i.p	2.35±0.04	85.31±0.04^{a}

ANFM; *A. nitida* fruit methanol extract, ANFH; *A. nitida* fruit n-hexane fraction, ANFC; *A. nitida* fruit chloroform fraction, ANFE; *A. nitida* fruit ethyl acetate fraction, ANFA; *A. nitida* fruit aqueous fraction. Values are presented as mean±SD (n=7). Means with different superscript $^{(a-d)}$ letters in column are significantly ($P < 0.01$) different from each other.

Table S9. Effect of *A. nitida* leaves and its fractions on hot plate test in rats

Group	Dose/route	Latency time in seconds/percentage analgesic activity			
		**0 min**	**30 min**	**60 min**	**120 min**
**Saline**	**2 ml, i.p**	7.07±0.19	7.03±0.08	7.01±0.12	6.76±0.20
**ANLM**	**50 mg/kg,p.o.**	7.07±0.16 (3.21±0.80^{a})	14.14±0.38 (38.56±1.89^{b})	14.86±1.14 (41.44±5.72^{b})	15.39±0.48 (43.38±2.42^{b})
	**100 mg/kg,p.o.**	7.06±0.15 (3.14±0.76^{a})	14.86±0.38 (42.14±1.89^{b})	15.71±0.49 (45.72±2.44^{b})	16.94±0.75 (51.16±3.73^{b})
	**200 mg/kg,p.o.**	7.04±0.11 (3.06±0.57^{a})	17.29±0.49 (54.28±2.44^{b})	17.79±0.70 (56.08±3.49^{b})	18.14±0.69 (57.16±3.45^{b})
**ANLH**	**50 mg/kg,p.o.**	7.09±0.23 (3.28±1.13^{a})	7.43±0.53 (4.99±2.67^{d})	9.86±0.40 (16.29±1.86^{c})	10.06±0.10 (16.69±0.42^{c})
	**100 mg/kg,p.o.**	7.09±0.38 (3.28±1.90^{a})	10.07±0.13 (18.21±0.63^{c})	10.39±0.36 (19.08±1.81^{d})	10.97±0.08 (21.31±0.38^{c})
	**200 mg/kg,p.o.**	7.04±0.45 (3.06±2.23^{a})	10.43±0.53 (19.99±2.67^{d})	10.71±0.49 (20.72±2.44^{d})	11.29±0.49 (22.88±2.44^{d})
**ANLC**	**50 mg/kg,p.o.**	7.03±0.22 (2.99±1.11^{a})	17.29±0.49 (54.28±2.44^{a})	18.04±0.31 (57.36±1.55^{a})	18.61±0.45 (59.52±2.24^{a})
	**100 mg/kg,p.o.**	7.16±0.23 (3.64±1.15^{a})	18.31±0.42 (59.42±2.09^{b})	18.96±0.11 (61.94±0.57^{a})	19.31±0.29 (63.02±1.43^{a})
	**200 mg/kg,p.o.**	7.09±0.15 (3.28±0.73a)	18.51±0.65 (60.42±3.26^{a})	19.27±0.31 (63.51±1.57^{a})	19.74±0.36 (65.16±1.80^{a})
**ANLE**	**50 mg/kg,p.o.**	7.01±0.35 (3.14±1.85^{a})	13.64±0.63 (36.06±3.13^{b})	14.19±0.63 (38.08±3.17^{b})	15.21±0.39 (42.52±1.97^{b})
	**100 mg/kg,p.o.**	7.03±0.35 (2.99±1.75^{a})	14.79±0.27 (41.78±1.37^{b})	15.16±0.27 (42.94±1.35^{bc})	16.31±0.39 (48.02±1.97^{b})
	**200 mg/kg,p.o.**	7.01±0.16 (2.92±0.79^{a})	16.57±0.53 (50.71±2.67^{bc})	17.33±0.42 (53.79±2.10^{b})	17.77±0.18 (55.31±0.90^{bc})
**ANLA**	**50 mg/kg,p.o.**	7.07±0.19 (3.21±0.94^{a})	12.57±0.53 (30.71±2.67^{c})	14.60±0.46 (40.15±2.31^{b})	14.93±0.64 (41.09±3.20^{b})
	**100 mg/kg,p.o.**	7.00±0.32	14.60±0.32	14.90±0.13	16.20±0.22

		(2.85±1.61[a])	(40.85±1.58[b])	(41.65±0.65[c])	(47.45±1.08[b])
	**200 mg/kg,p.o.**	7.06±0.20<br>(3.14±0.99[a])	15.94±0.10<br>(47.56±0.49[c])	16.37±0.36<br>(49.01±1.80[c])	17.17±0.37<br>(52.31±1.86[c])
**Morphine**	**(10 mg/kg)**	7.07±0.13<br>(3.21±0.63[a])	18.0±0.58<br>(57.85±2.89[a])	18.43±0.53<br>(63.29±2.67[a])	18.57±0.53<br>(69.31±2.67[a])
**Aspirin**	**(10 mg/kg)**	7.10±0.19<br>(3.35±0.96[a])	19.29±0.49<br>(64.28±2.44[a])	19.71±0.37<br>(65.72±1.84[a])	20.16±0.42<br>(67.24±2.08[a])

ANLM; *A. nitida* leaves methanol extract, ANLH; *A. nitida* leaves n-hexane fraction, ANLC; *A. nitida* leaves chloroform fraction, ANLE; *A. nitida* leaves ethyl acetate fraction, ANLA; *A. nitida* leaves aqueous fraction. Values are presented as mean±SD (n=7). Means with different superscript (a-g) letters in column are significantly ($P < 0.01$) different from each other. Percentage analgesic activity is shown in brackets.

Table S10. Effect of *A. nitida* fruit extract and its fractions on hot plate test in rats

Group	Dose/route	Latency time in seconds/percentage analgesic activity			
		**0 min**	**30 min**	**60 min**	**120 min**
**Saline**	**2 ml, i.p**	7.07±0.19	7.03±0.08	7.01±0.12	6.76±0.20
**ANFM**	**50 mg/kg,p.o.**	7.07±0.16 (3.21±0.80[a])	14.43±0.53 (39.99±2.67[c])	16.14±0.90 (47.86±4.50[c])	16.69±0.47 (49.88±2.37[c])
	**100 mg/kg,p.o.**	7.04±0.16 (3.06±0.81[ab])	15.71±0.49 (46.42±2.44[b])	16.74±0.73 (50.86±3.64[b])	17.37±0.48 (53.31±2.41[b])
	**200 mg/kg,p.o.**	6.97±0.19 (2.71±0.94[a])	16.11±0.30 (48.42±1.51[b])	17.29±0.49 (53.58±2.44[b])	17.86±0.90 (55.74±4.50[cd])
**ANFH**	**50 mg/kg,p.o.**	6.66±0.19 (1.14±0.95[b])	7.33±0.53 (4.49±2.66[e])	9.43±0.53 (14.29±2.67[e])	10.06±0.10 (16.69±0.42[e])
	**100 mg/kg,p.o.**	6.64±0.18 (1.06±0.91[c])	9.43±0.53 (14.99±2.67[d])	10.10±0.17 (17.65±0.87[d])	10.57±0.53 (19.31±2.67[d])
	**200 mg/kg,p.o.**	6.89±0.20 (2.28±0.98[a])	10.14±0.38 (18.56±1.89[d])	10.86±1.07 (21.44±5.35[d])	11.47±0.77 (23.81±3.84[e])
**ANFC**	**50 mg/kg,p.o.**	6.91±0.12 (2.42±0.61[ab])	16.29±0.49 (49.28±2.44[b])	17.44±0.50 (54.36±2.48[ab])	18.10±0.64 (56.95±3.18[ab])
	**100 mg/kg,p.o.**	6.97±0.13 (2.71±0.63[abc])	16.71±0.76 (51.42±3.78[b])	17.94±0.15 (56.86±0.76[a])	18.61±0.63 (59.52±3.17[a])
	**200 mg/kg,p.o.**	7.04±0.19 (3.06±0.95[a])	16.89±0.68 (52.28±4.16[b])	18.40±0.40 (59.15±2.31[b])	19.17±0.37 (62.31±1.86[ab])
**ANFE**	**50 mg/kg,p.o.**	6.77±0.17 (1.99±0.85[ab])	13.43±0.53 (34.99±2.67[d])	13.87±0.39 (36.51±1.93[d])	14.60±0.50 (39.45±2.52[d])
	**100 mg/kg,p.o.**	6.77±0.20 (1.71±0.99[abc])	13.64±0.27 (36.06±4.02[c])	14.57±0.53 (40.01±2.67[c])	16.07±0.13 (46.81±0.63[c])
	**200 mg/kg,p.o.**	6.90±0.16 (2.35±0.82[a])	14.43±0.98 (39.99±4.88[c])	15.29±0.76 (43.58±3.78[c])	16.70±0.83 (49.95±4.16[d])
**ANFA**	**50 mg/kg,p.o.**	7.09±0.23 (3.28±1.13[a])	15.26±0.64 (44.14±2.21[c])	16.57±0.53 (50.01±2.67[bc])	17.34±0.44 (53.16±2.21[bc])
	**100 mg/kg,p.o.**	6.71±0.20	16.14±0.69	17.00±0.58	17.71±0.71

		(1.42±0.98^{bc})	(48.56±3.45^{b})	(52.15±2.89^{b})	(55.02±3.54^{ab})
	**200 mg/kg,p.o.**	6.87±0.20 (2.21±0.99^{a})	16.24±0.52 (49.06±2.61^{b})	17.50±0.87 (54.65±4.33^{b})	18.43±0.53 (58.59±2.67^{bc})
**Morphine**	**(10 mg/kg)**	7.07±0.13 (3.21±0.63^{a})	18.0±0.58 (57.85±2.89^{a})	18.43±0.53 (63.29±2.67^{a})	18.57±0.53 (69.31±2.67^{a})
**Aspirin**	**(10 mg/kg)**	7.10±0.19 (3.35±0.96^{a})	19.29±0.49 (64.28±2.44^{a})	19.71±0.37 (65.72±1.84^{a})	20.16±0.42 (67.24±2.08^{a})

ANFM; *A. nitida* fruit methanol extract, ANFH; *A. nitida* fruit n-hexane fraction, ANFC; *A. nitida* fruit chloroform fraction, ANFE; *A. nitida* fruit ethyl acetate fraction, ANFA; *A. nitida* fruit aqueous fraction. Values are presented as mean±SD (n=7). Means with different superscript $^{(a-d)}$ letters in column are significantly ($P < 0.01$) different from each other. Percentage analgesic activity is shown in brackets.

Table S11. Effect of *A. nitida* leaves extract and its fractions on acetic acid induced writhing in rats

Groups	Dose/route	No. of writhing (mean ± SD)	% inhibition
**Saline**	**10 ml, i.p.**	71.33±3.21	0
**ANLM**	**50 mg/kg,p.o.**	28.02±1.11	$60.21±2.33^{ab}$
	**100 mg/kg,p.o.**	25.01±1.17	$64.47±2.17^{ab}$
	**200 mg/kg,p.o.**	22.00±2.30	$68.74±3.28^{a}$
**ANLH**	**50 mg/kg,p.o.**	62.05±3.41	$11.90±1.05^{f}$
	**100 mg/kg,p.o.**	58.04±2.33	$17.59±1.43^{f}$
	**200 mg/kg,p.o.**	52.28±3.14	$27.70±4.47^{e}$
**ANLC**	**50 mg/kg,p.o.**	21.00±1.61	$70.16±2.99^{a}$
	**100 mg/kg,p.o.**	15.22±1.14	$78.68±3.21^{a}$
	**200 mg/kg,p.o.**	12.93±0.04	$81.62±0.06^{a}$
**ANLE**	**50 mg/kg,p.o.**	49.00±2.33	$30.37±2.22^{de}$
	**100 mg/kg,p.o.**	45.01±2.92	$36.06±1.45^{d}$
	**200 mg/kg,p.o.**	42.29±0.29	$39.90±0.41^{d}$
**ANLA**	**50 mg/kg,p.o.**	33.01±1.77	$53.11±3.35^{c}$
	**100 mg/kg,p.o.**	29.03±1.08	$58.79±3.11^{bc}$
	**200 mg/kg,p.o.**	25.57±2.14	$62.24±3.05^{ab}$
**Aspirin**	**10 mg/kg,i.p.**	20.13±1.12	$71.39±2.00^{a}$
**Morphine**	**10 mg/kg,i.p.**	13.22±1.89	$81.65±2.14^{a}$

ANLM; *A. nitida* leaves methanol extract, ANLH; *A. nitida* leaves n-hexane fraction, ANLC; *A. nitida* leaves chloroform fraction, ANLE; *A. nitida* leaves ethyl acetate fraction, ANLA; *A. nitida* leaves aqueous fraction. Values are presented as mean±SD (n=7). Means with different superscript $^{(a-g)}$ letters in column are significantly ($P < 0.01$) different from each other. Percentage analgesic activity is shown in brackets.

Table S12. Effect of *A. nitida* fruit extract and its fractions on acetic acid induced writhing in rats

Groups	Dose/route	No. of writhing (mean ± SD)	% inhibition
**Saline**	**10 ml, i.p.**	71.33±3.21	0
**ANFM**	**50 mg/kg,p.o.**	38.01±0.27	50.11 ± 3.35^{cd}
	**100 mg/kg,p.o.**	35.03±1.08	53.79 ± 2.11^{cd}
	**200 mg/kg,p.o.**	31.57±2.24	58.24 ± 3.05^{b}
**ANFH**	**50 mg/kg,p.o.**	63.01±3.11	10.20 ± 1.25^{f}
	**100 mg/kg,p.o.**	57.04±2.33	18.99 ± 1.43^{f}
	**200 mg/kg,p.o.**	57.28±3.19	24.70 ± 4.47^{e}
**ANFC**	**50 mg/kg,p.o.**	28.00±0.61	65.16 ± 2.99^{a}
	**100 mg/kg,p.o.**	23.22±1.24	72.68 ± 2.21^{a}
	**200 mg/kg,p.o.**	16.93±0.14	75.62 ± 2.06^{a}
**ANFE**	**50 mg/kg,p.o.**	48.00±2.33	30.37 ± 2.22^{de}
	**100 mg/kg,p.o.**	46.01±1.92	35.06 ± 1.05^{d}
	**200 mg/kg,p.o.**	41.29±1.29	39.90 ± 0.41^{d}
**ANFA**	**50 mg/kg,p.o.**	32.02±1.21	55.21 ± 2.40^{bc}
	**100 mg/kg,p.o.**	30.01±1.18	60.47 ± 2.20^{ab}
	**200 mg/kg,p.o.**	28.00±2.50	64.74 ± 3.18^{ab}
**Aspirin**	**10 mg/kg,i.p.**	20.13±1.12	71.39 ± 2.00^{a}
**Morphine**	**10 mg/kg,i.p.**	13.22±1.89	81.65 ± 2.14^{a}

ANFM; *A. nitida* fruit methanol extract, ANFH; *A. nitida* fruit n-hexane fraction, ANFC; *A. nitida* fruit chloroform fraction, ANFE; *A. nitida* fruit ethyl acetate fraction, ANFA; *A. nitida* fruit aqueous fraction. Values are presented as mean±SD (n=7). Means with different superscript $^{(a-d)}$ letters in column are significantly ($P < 0.01$) different from each other. Percentage analgesic activity is shown in brackets.
